# Regulatory effects of mepiquat chloride on soybean yield formation and physiological responses to drought stress

**DOI:** 10.3389/fpls.2026.1923691

**Published:** 2026-09-07

**Authors:** Xinyu Zhou, Xiaomei Li, Wei Zhao, Xiyue Wang, Yuanqi Ma, Shoukun Dong

**Affiliations:** 1 College of Agriculture, Northeast Agricultural University, Harbin, China; 2 College of Agriculture, Heilongjiang Agricultural Engineering Vocational Technical University, Harbin, China

**Keywords:** coordinated root-leaf regulation, drought stress, endogenous hormones, mepiquat chloride, phenylpropanoid metabolism, soybean

## Abstract

Mepiquat chloride (DPC) regulates crop architecture, but its effects on soybean yield formation and drought-related physiological responses remain insufficiently understood. We conducted field experiments to assess the effects of five DPC concentrations (0, 125, 200, 275, and 500 mg L^−1^) on plant architecture, yield components, and grain yield in two soybean cultivars with contrasting drought tolerance, Heinong 44 (HN44) and Heinong 65 (HN65). We then used a sand-culture experiment with PEG-6000-induced osmotic stress to examine DPC-associated changes in root and shoot growth and stress-related physiology. Under field conditions, DPC reduced plant height and internode elongation and increased stem diameter. It also altered pod-set composition and increased seed number per plant, resulting in higher grain yield. The greatest yield increase in HN44 occurred at 200 mg L^−1^ DPC (29.44%), whereas the largest numerical increase in HN65 occurred at 275 mg L^−1^ DPC (12.99%). However, yield did not differ significantly between the 200 and 275 mg L^−1^ treatments in HN65; therefore, 200 mg L^−1^ DPC was selected for the subsequent osmotic-stress experiment. Under PEG-induced osmotic stress, DPC restricted shoot and leaf-area expansion while promoting root growth and biomass accumulation. DPC-treated plants maintained higher SPAD values and PSII-related fluorescence parameters and showed lower non-photochemical quenching. In leaves and roots, DPC treatment was associated with lower hydrogen peroxide and malondialdehyde contents, higher antioxidant-enzyme activities, and greater osmotic-adjustment capacity. DPC treatment was also associated with enhanced phenylpropanoid metabolism, greater lignin accumulation, and tissue-specific changes in endogenous hormone profiles. These findings show that DPC can improve yield formation under the non-drought field conditions examined and modulate morphological and physiological responses under PEG-induced osmotic stress. They provide a basis for optimizing DPC application in soybean production and for further evaluating its role in drought adaptation.

## 1 Introduction

Soybean (*Glycine max* (L.) Merr.) is a major source of plant protein and oil for food and feed and is central to global agricultural supply chains (Uskutoğlu, 2025). As demand for soybean continues to increase, improving yield per unit area and maintaining stable production on limited arable land are increasingly important (Pagano and Miransari, 2016). Climate warming has also increased soybean exposure to abiotic stresses, including drought, salinity–alkalinity, and extreme temperatures (Sabagh et al., 2019; Zhu et al., 2024). Among these stresses, drought is a major constraint on high and stable soybean yield because of its frequency and broad geographic extent (Shaheen et al., 2016).

Drought-induced water deficit inhibits soybean growth, reducing plant height and leaf area and impairing root development. These effects can weaken plant water uptake and disrupt cellular homeostasis. Drought also promotes the accumulation of reactive oxygen species (ROS), increases membrane lipid peroxidation, and impairs light capture and photosynthetic electron transport, ultimately limiting photosynthate production (Kulundžić et al., 2022; Jan et al., 2025; Huang et al., 2026; Wahab et al., 2022). Plants counter these effects through coordinated defense responses, including activation of antioxidant enzymes to remove excess ROS and accumulation of osmotic-adjustment substances to maintain cellular water status. Endogenous hormones also coordinate growth, development, and stress responses (Ullah et al., 2018; Wahab et al., 2022).

Plant growth regulators are widely used to modify crop architecture, improve yield, and enhance tolerance to abiotic stress. Mepiquat chloride (DPC) is a systemic plant growth retardant that suppresses gibberellin (GA) biosynthesis and thereby restricts excessive cell elongation (Tung et al., 2020). At appropriate concentrations, DPC can reduce plant height, compact plant architecture, and increase stem diameter. It can also promote dry-matter allocation to seeds or fruits and increase final yield (Tung et al., 2018; Shi et al., 2022). In addition to regulating morphology and yield, DPC has been reported to maintain cellular structural integrity and metabolic stability under adverse conditions, thereby mitigating drought-induced damage (Wu et al., 2023). However, studies of DPC-mediated stress responses in soybean have mainly examined individual traits or organs, such as shoot architecture, leaf photosynthesis, antioxidant capacity, or root growth. Evidence remains limited on coordinated aboveground and belowground responses to DPC under osmotic stress, particularly the integration of physiological traits, phenylpropanoid metabolism, and endogenous hormone profiles.

We therefore examined two soybean cultivars with contrasting drought tolerance, Heinong 44 (HN44) and Heinong 65 (HN65). Previous drought-resistance screening identified HN44 as relatively drought tolerant and HN65 as relatively drought sensitive (Wang et al., 2012). We first conducted a field experiment under natural, non-drought conditions to evaluate the effects of different DPC concentrations on plant architecture, yield components, and grain yield. Based on these results, 200 mg L^−1^ DPC was selected for a separate sand-culture experiment because it provided stable agronomic performance across both cultivars. The sand-culture experiment used PEG-6000-induced osmotic stress to assess DPC-associated morphological, physiological, phenylpropanoid-metabolic, and endogenous-hormone responses in shoots and roots. This study provides an integrated assessment of soybean responses associated with DPC treatment under osmotic stress and may inform its agronomic use in soybean production.

## 2 Materials and methods

### 2.1 Plant materials and experimental site

Two soybean cultivars with contrasting drought tolerance were used: Heinong 44 (HN44), which is relatively drought tolerant, and Heinong 65 (HN65), which is relatively drought sensitive (Wang et al., 2012). Seeds were supplied by the Heilongjiang Academy of Agricultural Sciences. The tested plant growth regulator was mepiquat chloride (DPC), supplied as a 98% soluble-powder formulation by Henan Zhongwei Chunyu Plant Nutrition Co., Ltd. (China).

The field trial was conducted from May to October 2024 at the Xiangyang Experimental Base, Harbin, Heilongjiang Province, China. No drought treatment was imposed in the field experiment. Daily air temperature and precipitation from 1 May to 15 October 2024 are shown in **Supplementary Figure 1**. The site has a mid-temperate continental monsoon climate and black soil. The main physicochemical properties of the experimental soil are listed in **Supplementary Table 1**. The pot experiment was conducted in 2025 at Northeast Agricultural University, Harbin, China, to evaluate DPC-associated responses to PEG-induced osmotic stress under controlled conditions.

### 2.2 Experimental design

#### 2.2.1 Field experiment

The field experiment used a two-factor factorial design with three independent biological replicates. The factors were cultivar, HN44 or HN65, and DPC concentration, 0, 125, 200, 275, or 500 mg L^−1^. The resulting 10 treatment combinations were randomly assigned to plots, with three plots per combination (30 plots in total). Each plot comprised eight 5-m ridges spaced 0.65 m apart and had an area of 26.0 m². Each plot was considered one independent biological replicate. DPC was applied by foliar spraying at the V3 stage until leaves were wetted without runoff; a second application was made 1 week later. Field management followed local conventional soybean-production practices, including hand weeding and pest and disease control. At R8, one centrally located 2m² sampling area was harvested from each plot, excluding border rows, to determine morphological traits, yield components, and grain yield.

#### 2.2.2 Pot experiment

Based on field measurements of plant architecture, yield components, grain yield, and principal component analysis, 200 mg L^−1^ DPC (D200) was selected for the subsequent sand-culture experiment. Although the highest numerical grain yield of HN65 occurred at 275 mg L^−1^ DPC (D275), grain yield and seed number per plant did not differ significantly between D200 and D275. In contrast, HN44 showed its highest grain yield at D200. D200 was therefore selected because it provided stable agronomic performance across both cultivars while avoiding the potential growth inhibition associated with higher DPC concentrations. The subsequent experiment evaluated DPC-associated morphological and physiological responses under PEG-induced osmotic stress.

Plastic pots with drainage holes (30 cm high and 28 cm in diameter) were lined with gauze and filled with washed river sand. Full, uniform, pest-free soybean seeds were sown in each pot. From emergence until unifoliate-leaf expansion, each pot received 500 mL clean water daily. When the unifoliate leaves had fully expanded, seedlings were thinned to three uniform plants per pot. Pots subsequently received 500 mL Hoagland nutrient solution daily (composition in **Supplementary Table 2**). Treatments began when the third trifoliate leaf had fully expanded (V3). The following treatments were established: (i) CK, foliar application of water and daily irrigation with 500 mL standard Hoagland nutrient solution; (ii) DS, foliar application of water and daily irrigation with 500 mL Hoagland nutrient solution containing 10% PEG-6000; and (iii) DS + D200, foliar application of 200 mg L^−1^ DPC and daily irrigation with 500 mL Hoagland nutrient solution containing 10% PEG-6000. Foliar applications were made until leaves were wetted without runoff (approximately 75 mL pot−1), and a second application was made 3 d after the first. Samples were collected between 08:00 and 09:00 at 3, 9, and 15 d after the second application. Six pots were sampled per treatment, each representing an independent biological replicate. Three pots (n = 3) were used for physiological measurements. The second fully expanded leaf from the apex and washed, blotted roots were collected from the three plants in each pot, immediately frozen in liquid nitrogen, and stored at −80 °C. Samples from the three plants within a pot were pooled to form one composite sample for physiological analysis. The remaining three pots (n = 3) were used to measure shoot and root morphological traits.

### 2.3 Measurements and methods

#### 2.3.1 Field morphology and yield measurements

At full maturity (R8), 10 plants representing the growth status of each plot were randomly selected to measure morphological traits and yield components. Plant height was measured from the cotyledonary node to the apical growing point. Stem diameter, internode length, pod number per plant, and seed number per plant were also recorded. Grain yield was determined by manually harvesting one centrally located 2m² area from each plot, excluding border rows. Each plot was treated as one independent biological replicate and as the experimental unit for statistical analysis (n = 3 per treatment combination).

#### 2.3.2 Pot morphology and biomass measurements

Plant height was measured with a ruler. Shoot and root fresh weights were recorded immediately after sampling. Root traits were quantified after scanning with a Phantom 9900XL root scanner (Microtek, China). Total root length, root surface area, mean root diameter, and root volume were calculated using WinRHIZO v2019 software. Leaf area was measured for each plant with a Yaxin-1241 leaf-area meter (Beijing Yaxin, China). For dry-matter determination, samples were heated at 105 °C for 30 min and then dried at 65 °C to constant weight.

#### 2.3.3 Chlorophyll fluorescence parameter measurements

Chlorophyll fluorescence was measured on the second fully expanded leaf from the apex using a MultispeQ V2.0 plant phenotyping device (PhotosynQ Inc., East Lansing, MI, USA), following Kuhlgert et al. (2016). The Photosynthesis RIDES 2.0 protocol was used to measure the maximum photochemical efficiency of photosystem II under light, the non-photochemical quenching coefficient, the actual photochemical efficiency of photosystem II, and relative chlorophyll content. Three biological replicates were measured per treatment. Two plants were assessed in each pot, and two technical readings per plant were averaged.

#### 2.3.4 Stress-resistance physiology and osmotic regulatory substances

Hydrogen peroxide content was determined by the potassium iodide method, and malondialdehyde content was measured using the thiobarbituric acid reaction (Schmedes and Hølmer, 1989; Junglee et al., 2014). Superoxide dismutase, peroxidase, and ascorbate peroxidase activities were determined by nitroblue tetrazolium photoreduction, guaiacol oxidation, and ascorbate oxidation, respectively, following Li (2010). Proline was quantified by the acid-ninhydrin method. Soluble protein was determined using Coomassie Brilliant Blue G-250, and soluble sugar was measured by the anthrone colorimetric method (Wang and Huang, 2006a, 2006b; Ábrahám et al., 2010). For all antioxidant, oxidative-damage, and osmotic-adjustment measurements, three independent biological replicates were analysed per treatment (n = 3). Each biological replicate comprised one pooled sample from a single pot.

#### 2.3.5 Phenylpropanoid metabolism measurements

For phenylpropanoid-metabolism measurements, three independent biological replicates were analysed per treatment (n = 3), with one pooled sample from a single pot representing each replicate. Lignin content and the activities of phenylalanine ammonia-lyase and cinnamyl alcohol dehydrogenase were measured using commercial assay kits from Beijing Solarbio Science & Technology Co., Ltd. (China), with catalogue numbers BC4205, BC0215, and BC4175, respectively. All assays were conducted according to the manufacturer’s instructions. The activities of 4-coumarate:CoA ligase and trans-cinnamate 4-monooxygenase were determined using double-antibody sandwich enzyme-linked immunosorbent assay kits from Jiangsu Meimian Industrial Co., Ltd. (China), and absorbance was measured at 450 nm.

#### 2.3.6 Determination of endogenous hormone contents

Endogenous hormones were analysed in leaf and root samples collected 9 d after the second application, when the drought response was most evident. For each treatment and tissue, three independent biological replicates were analysed (n = 3), with one pooled sample from a single pot representing each replicate. Hormone profiling was performed by high-performance liquid chromatography coupled with tandem mass spectrometry (Pan et al., 2010; Niu et al., 2014). The quantified compounds included abscisic acid; the auxin-related metabolites L-tryptophan, tryptamine, methyl indole-3-acetate, indole-3-acetic acid, indole-3-acetylaspartic acid, and indole-3-acetylglutamic acid; the cytokinins isopentenyladenosine 5′-monophosphate and isopentenyladenosine; and the jasmonate-related compounds 12-oxo-phytodienoic acid, jasmonic acid, and jasmonoyl-isoleucine.

### 2.4 Data processing and statistical analysis

Data were compiled in Microsoft Excel 2010 and analysed using IBM SPSS Statistics 21.0 (IBM Corp., Armonk, NY, USA). Statistical significance was assessed at α = 0.05. Before analysis of variance, normality was evaluated using the Shapiro–Wilk test and homogeneity of variances using Levene’s test. Treatment means were compared using Duncan’s multiple range test at p < 0.05. Multifactorial analysis of variance was used to assess the main and interaction effects of experimental factors on the measured variables. Statistical plots and correlation heatmaps were prepared in Origin 9 (OriginLab Corp., Northampton, MA, USA). The conceptual response diagram was prepared in Adobe Illustrator 2026 (v30.0; Adobe Inc., San Jose, CA, USA).

## 3 Results

### 3.1 Effects of field DPC application on soybean plant height and stem diameter

**Figure 1:**
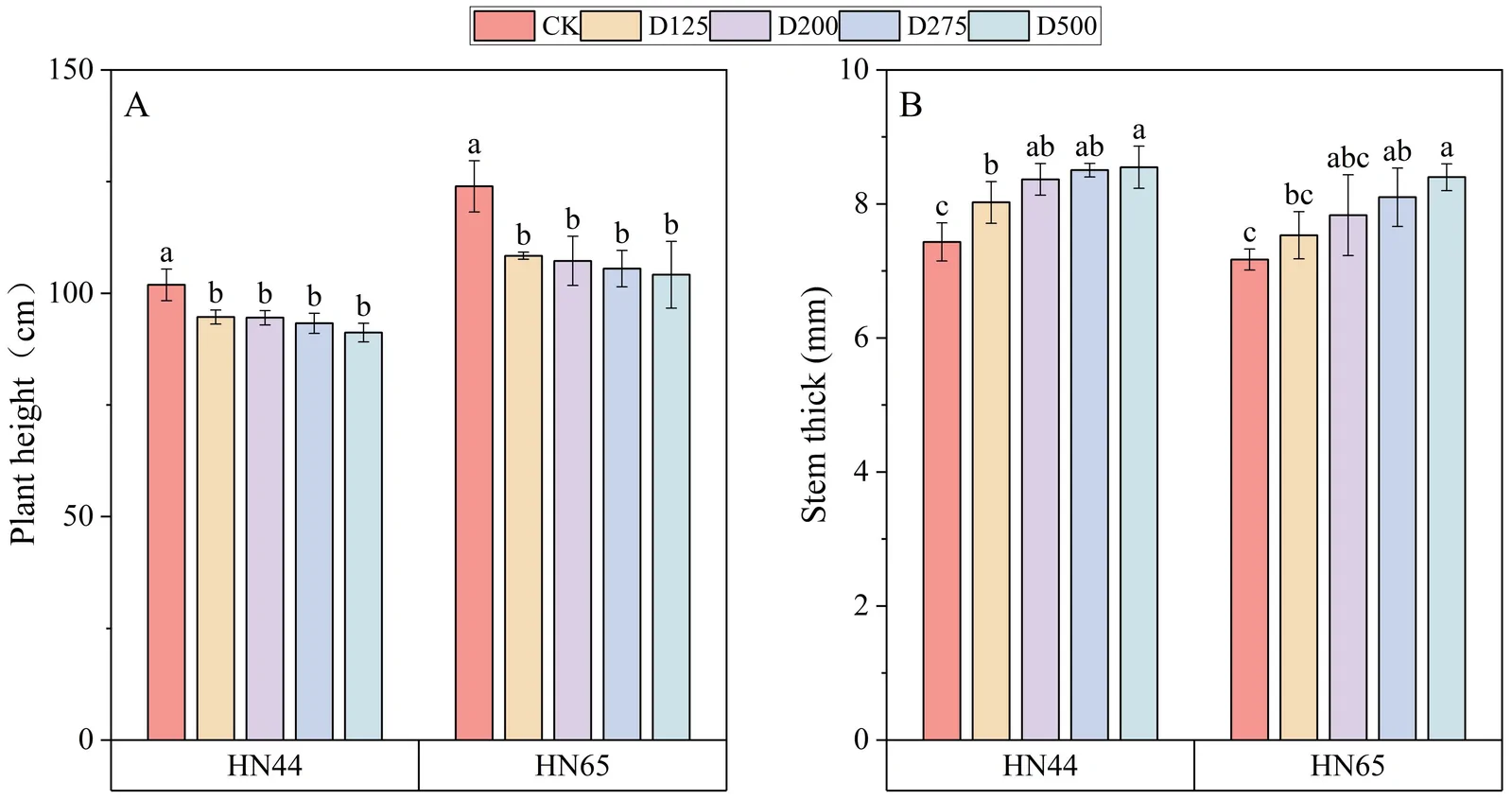
Effects of field DPC application on soybean plant height and stem diameter. **(A)** Plant height; **(B)** Stem diameter. Data are presented as means ± SD (n = 3). Different lowercase letters indicate significant differences among DPC treatments within the same cultivar according to Duncan’s multiple range test (P < 0.05). CK, control; D125, D200, D275, and D500 indicate DPC spraying concentrations of 125, 200, 275, and 500 mg L^-^¹, respectively.

As shown in **Figure 1**, DPC application altered soybean plant architecture by reducing plant height and increasing stem diameter. Plant height decreased significantly in both cultivars as DPC concentration increased (**Figure 1A**). Under D500, plant height decreased by 12.34% in HN44 and by 12.19% in HN65 relative to CK. In contrast, stem diameter increased with DPC concentration in both cultivars and was greatest under D500 (**Figure 1B**). Relative to CK, stem diameter increased by 15.01% in HN44 and by 17.15% in HN65.

### 3.2 Effects of field DPC application on soybean internode length and internode number

**Figure 2:**
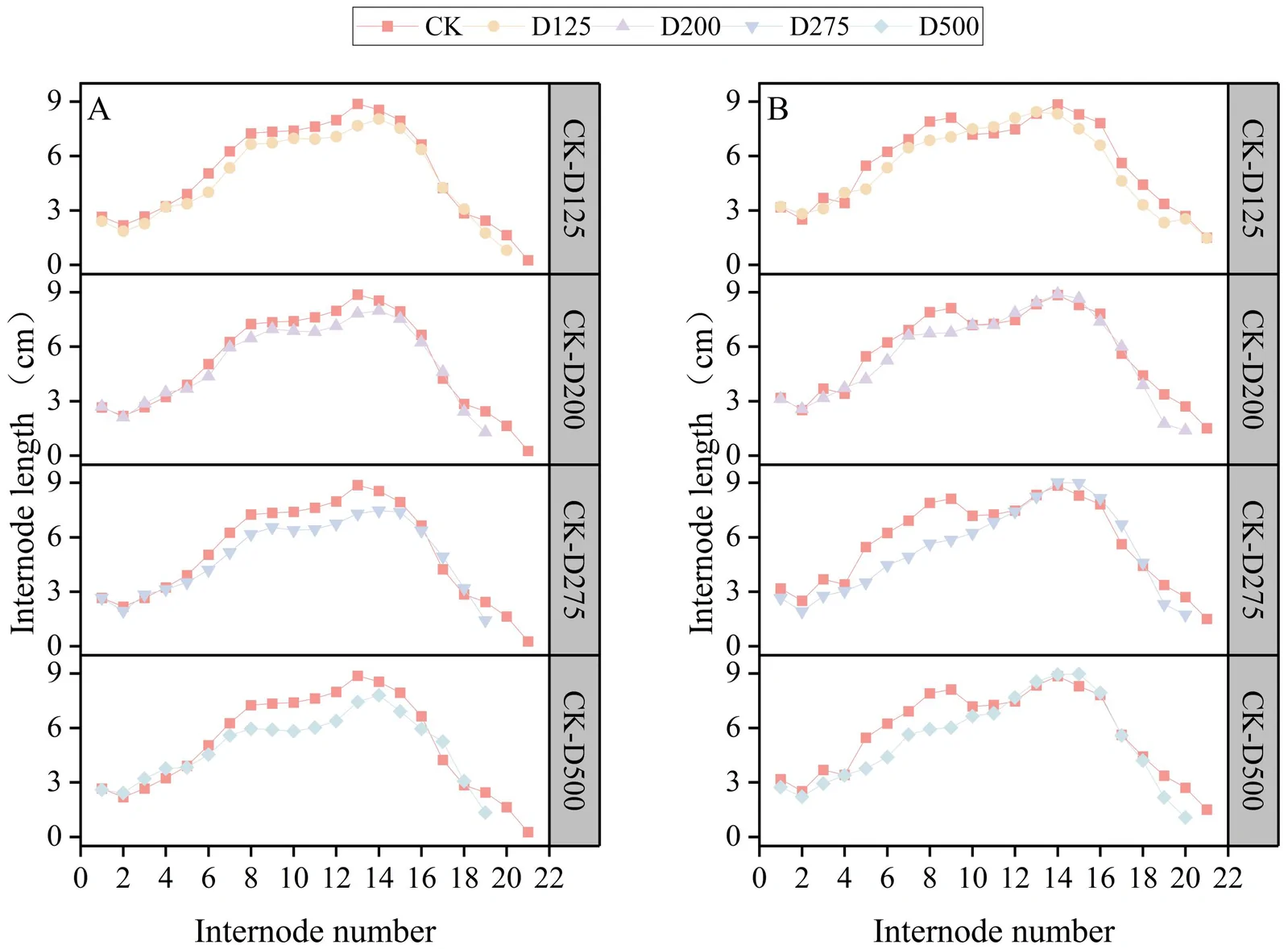
Effects of field DPC application on soybean internode length and internode number. **(A)** Internode length and total internode number in HN44; **(B)** Internode length and total internode number in HN65. Values are presented as means ± SD (n = 3). Statistical comparisons were conducted among DPC treatments within each cultivar using Duncan’s multiple range test (P < 0.05). CK, control; D125, D200, D275, and D500 indicate DPC spraying concentrations of 125, 200, 275, and 500 mg L^-^¹, respectively.

As shown in **Figure 2**, DPC application affected internode length and total internode number in soybean. Under CK, the cultivars showed distinct internode-length profiles. In HN44, internode length increased and then decreased with node number, and the longest internodes occurred at nodes 13–14. In HN65, internode length showed a bimodal pattern, with peaks at nodes 9 and 14. Under D125–D500, the decline in internode length at nodes 10–11 observed in CK-treated HN65 was not apparent, and internode length increased from node 9 to node 14.

Despite these cultivar-specific profiles, DPC significantly inhibited internode elongation in both cultivars, with stronger inhibition at higher concentrations. The response in HN44 was most apparent at nodes 6–14, whereas that in HN65 was concentrated at nodes 5–10. DPC application also reduced total internode number. Relative to CK, HN44 had one fewer internode under D125 and two fewer internodes under the remaining DPC treatments. HN65 had one fewer internode under D200, D275, and D500.

### 3.3 Effects of field DPC application on soybean pod number

**Figure 3:**
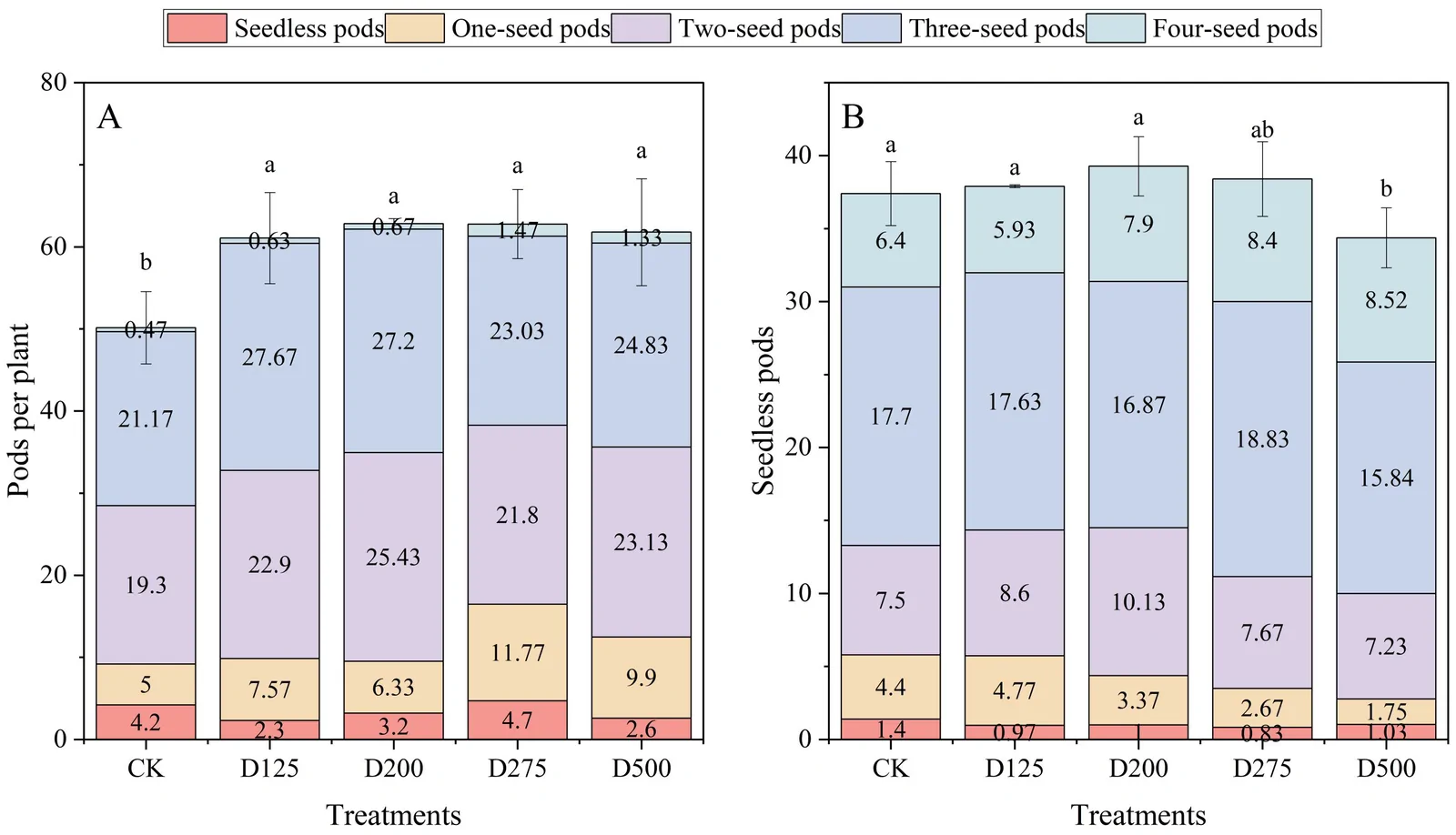
Effects of field DPC application on soybean pod number. **(A)** Number of pods per plant for HN44; **(B)** Number of pods per plant for HN65. Data are presented as means ± SD (n = 3). Different lowercase letters indicate significant differences in total pod number among DPC treatments within the same cultivar according to Duncan’s multiple range test (P < 0.05). CK, control; D125, D200, D275, and D500 indicate DPC spraying concentrations of 125, 200, 275, and 500 mg L^-^¹, respectively.

As shown in **Figure 3**, DPC application altered total pod number per plant and the distribution of pods with different seed numbers, with different responses between cultivars. Relative to CK, all DPC treatments increased total pod number per plant in HN44 by 21.80%–25.31%, although the DPC concentrations did not differ significantly from one another. In HN65, total pod number did not differ significantly under D125–D275, with increases of 1.34%–5.00%, but decreased significantly by 11.4% under D500 relative to CK.

DPC application also altered the distribution of pod classes in both cultivars. In HN44, the increase in total pod number was mainly associated with increases in two-seed and three-seed pods. The numbers of two-seed and three-seed pods were greatest under D200 and D125, increasing by 31.78% and 30.71%, respectively, relative to CK. D275 and D500 also increased four-seed pod number in HN44. In HN65, all DPC concentrations reduced ineffective pod number, although higher concentrations reduced total pod number. Four-seed pod number increased with DPC concentration and was 31.3% and 33.1% greater under D275 and D500, respectively, than under CK.

### 3.4 Effects of field DPC application on yield and yield components

**Figure 4:**
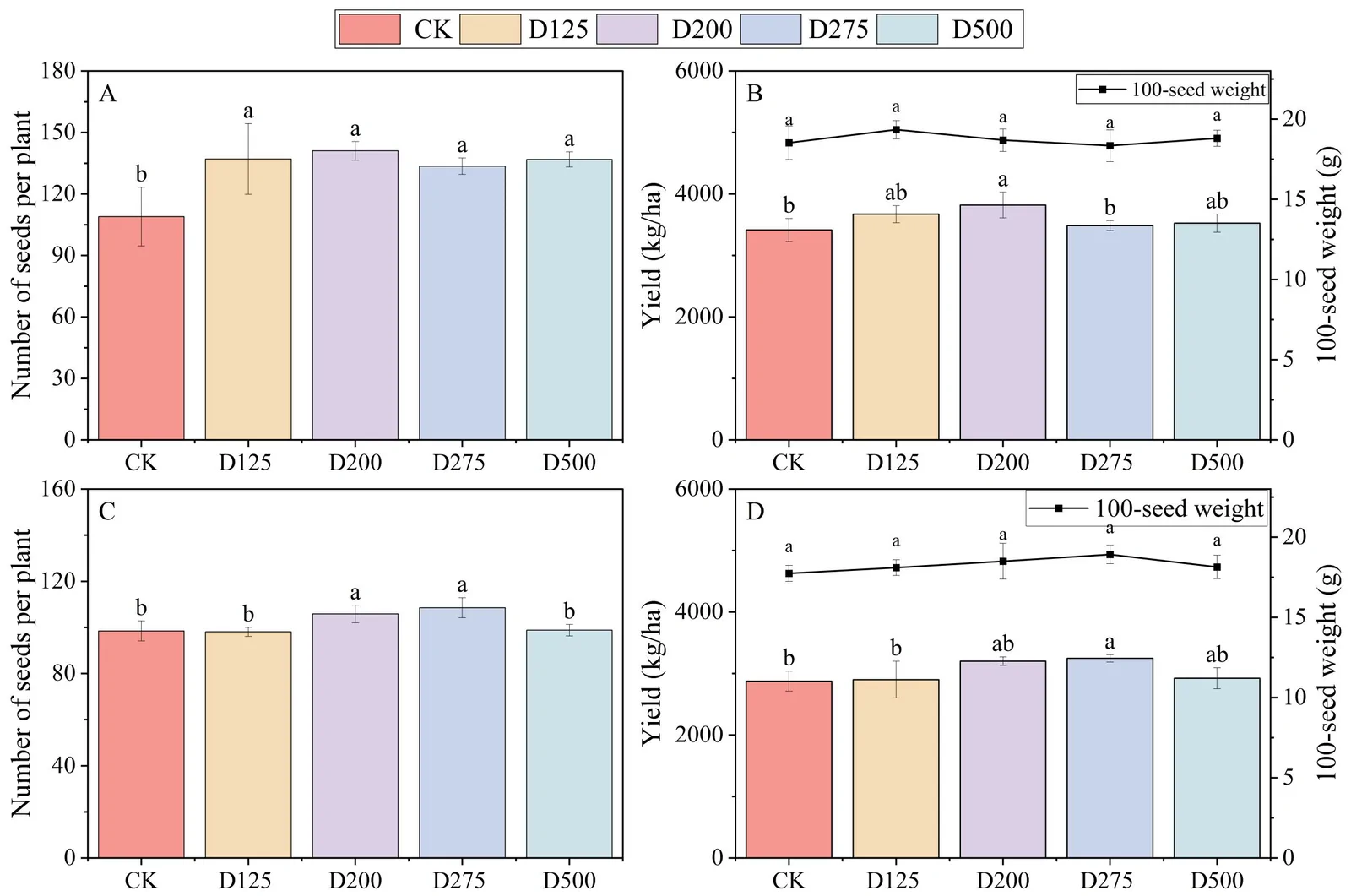
Effects of field DPC application on soybean yield components. **(A, B)** Seed number per plant, grain yield, and 100-seed weight in HN44; **(C, D)** seed number per plant, grain yield, and 100-seed weight in HN65. Data are presented as means ± SD (n = 3). Different lowercase letters indicate significant differences among DPC treatments within the same cultivar according to Duncan’s multiple range test (P < 0.05). CK, control; D125, D200, D275, and D500 indicate DPC spraying concentrations of 125, 200, 275, and 500 mg L^-^¹, respectively.

Field DPC application affected grain yield and yield components in both cultivars (**Figure 4**). DPC treatment increased grain yield and seed number per plant but did not significantly affect 100-seed weight. In HN44, seed number per plant and grain yield increased and then decreased as DPC concentration increased. Both variables were greatest under D200, increasing by 11.85% and 29.44%, respectively, relative to CK. In HN65, seed number per plant and grain yield were greatest under D275, increasing by 10.18% and 12.99%, respectively, relative to CK. However, neither variable differed significantly between D200 and D275; the numerical differences were 2.74% for seed number per plant and 1.63% for grain yield.

### 3.5 Correlation analysis and principal component analysis

**Figure 5:**
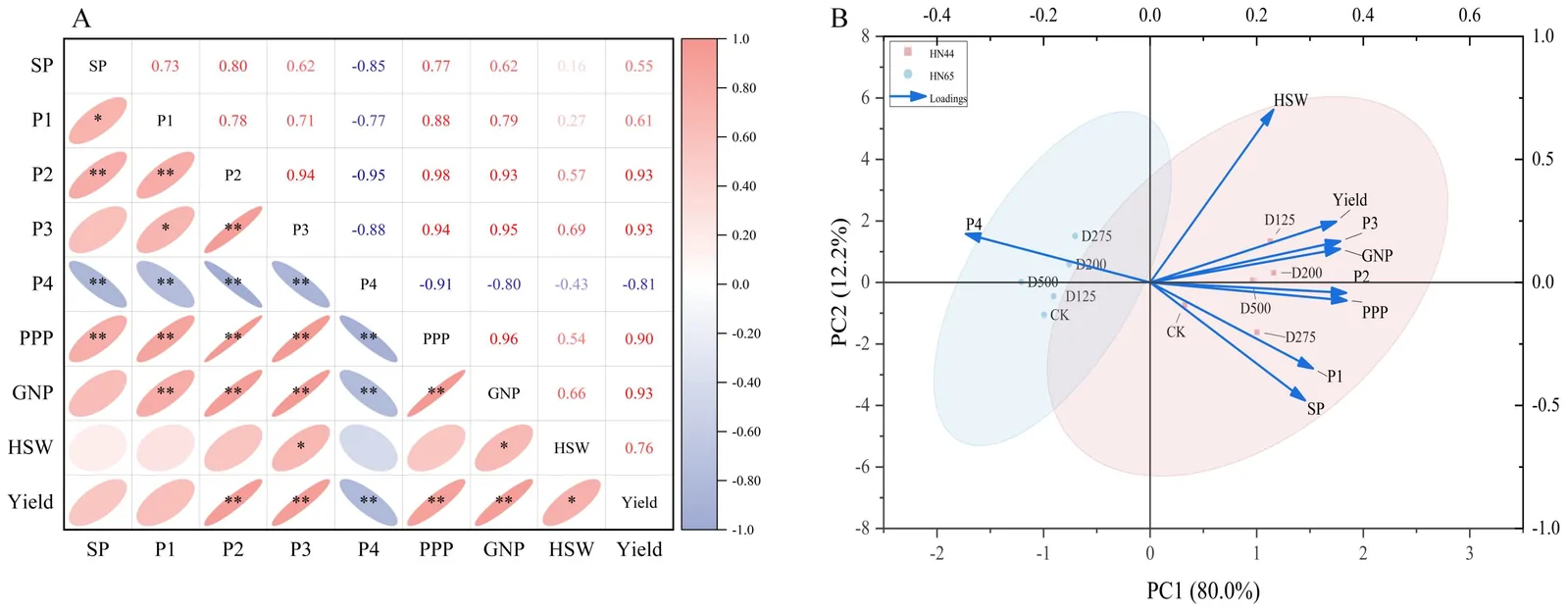
Correlation and principal component analyses of yield and yield-related traits. **(A)** Pearson correlation matrix among grain yield and yield-related traits. Red and blue indicate positive and negative correlations, respectively. * and ** indicate significance at P < 0.05 and P < 0.01, respectively. **(B)** Principal component analysis of grain yield, yield-related traits, cultivars, and DPC treatments. Arrows represent variable loadings, and points represent treatment scores. Yield, grain yield; PPP, seeds per plant; GNP, total pod number per plant; SP, ineffective pods; P1, one-seed pods; P2, two-seed pods; P3, three-seed pods; P4, four-seed pods; HSW, 100-seed weight.

To further investigate the regulatory effects of DPC on yield in the two soybean cultivars, correlation and principal component analyses were performed for yield components (**Figure 5**). As shown in **Figure 5A**, yield was extremely significantly and positively correlated with seed number per plant, total pod number per plant, three-seed pod number, and two-seed pod number (P < 0.01), with correlation coefficients all greater than 0.90. The 100-seed weight was also significantly positively correlated with yield (P < 0.05, r = 0.76), but the strength of this correlation was lower than those for seed number and pod-number traits. These results indicate that DPC increased yield mainly by increasing pod number per plant, especially the numbers of three-seed and two-seed pods, rather than by increasing seed weight. In addition, four-seed pod number was extremely significantly negatively correlated with yield and most other traits (P < 0.01), suggesting that the formation of four-seed pods may compete for nutrients to some extent, or that an increase in their number may not be effectively converted into final yield improvement.

In the PCA, PC1 and PC2 explained 80.0% and 12.2% of the total variation, respectively, for a cumulative contribution of 92.2% (**Figure 5B**). Grain yield, seed number per plant, three-seed pod number, two-seed pod number, and total pod number per plant had high positive loadings on PC1. Four-seed pod number was positioned on the negative side of PC1, whereas 100-seed weight was mainly associated with PC2. Relative to CK, HN44 shifted toward the positive side of PC1 under all DPC treatments, with the greatest shift under D200. The D500 score was lower than the D200 score. HN65 had its highest score under D275, whereas its pod-setting traits and grain yield under D200 were also significantly greater than those under CK. Based on these responses, D200 was selected for the subsequent PEG-induced drought-stress experiment. In HN65, grain yield and seed number per plant did not differ significantly between D200 and D275, whereas HN44 had its greatest grain yield and seed number per plant under D200.

### 3.6 Regulatory effects of field DPC application on soybean yield improvement

**Figure 6:**
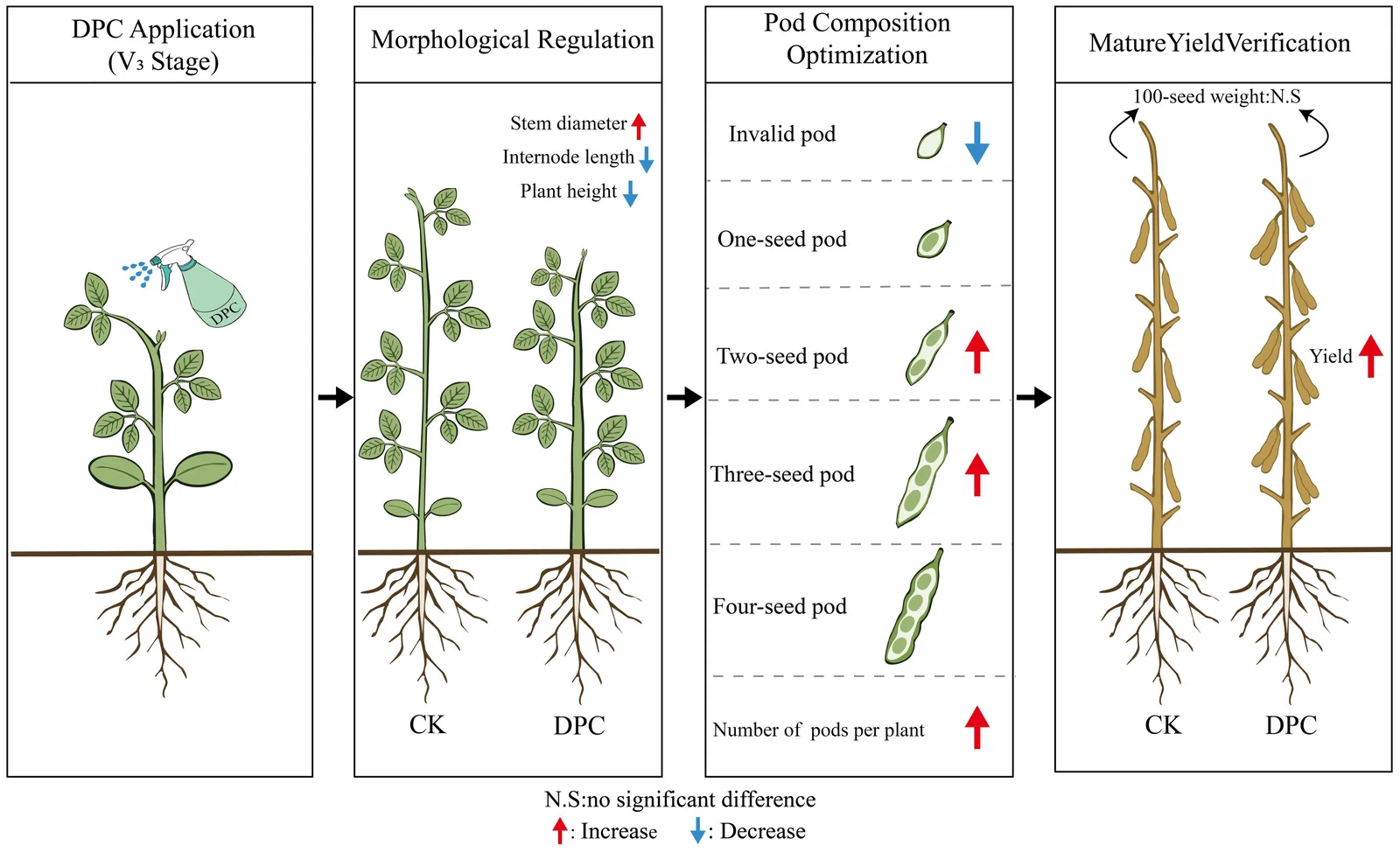
Regulatory effects of field DPC application on soybean yield improvement.

Together, the above results revealed the regulatory pathway by which field DPC application increased soybean yield (**Figure 6**). After DPC spraying at the V3 stage, plants underwent significant morphological remodeling, characterized by reduced plant height and internode length and increased stem diameter. These changes not only enhanced lodging resistance but also shortened the physical distance for transporting photosynthetic assimilates to sink organs. This morphological change further promoted the redistribution of photosynthates and optimized pod-setting structure, as evidenced by a significant reduction in ineffective pods and large increases in the proportions of two-seed and three-seed pods, thereby significantly increasing total effective pod number and seed number per plant. Yield verification at maturity showed that DPC significantly increased soybean yield, whereas 100-seed weight was not significantly affected. This indicates that DPC increased yield mainly by increasing seed number per plant through optimizing pod composition and increasing effective pod number, rather than by increasing seed weight.

### 3.7 Effects of DPC application on soybean morphology under drought stress

**Figure 7:**
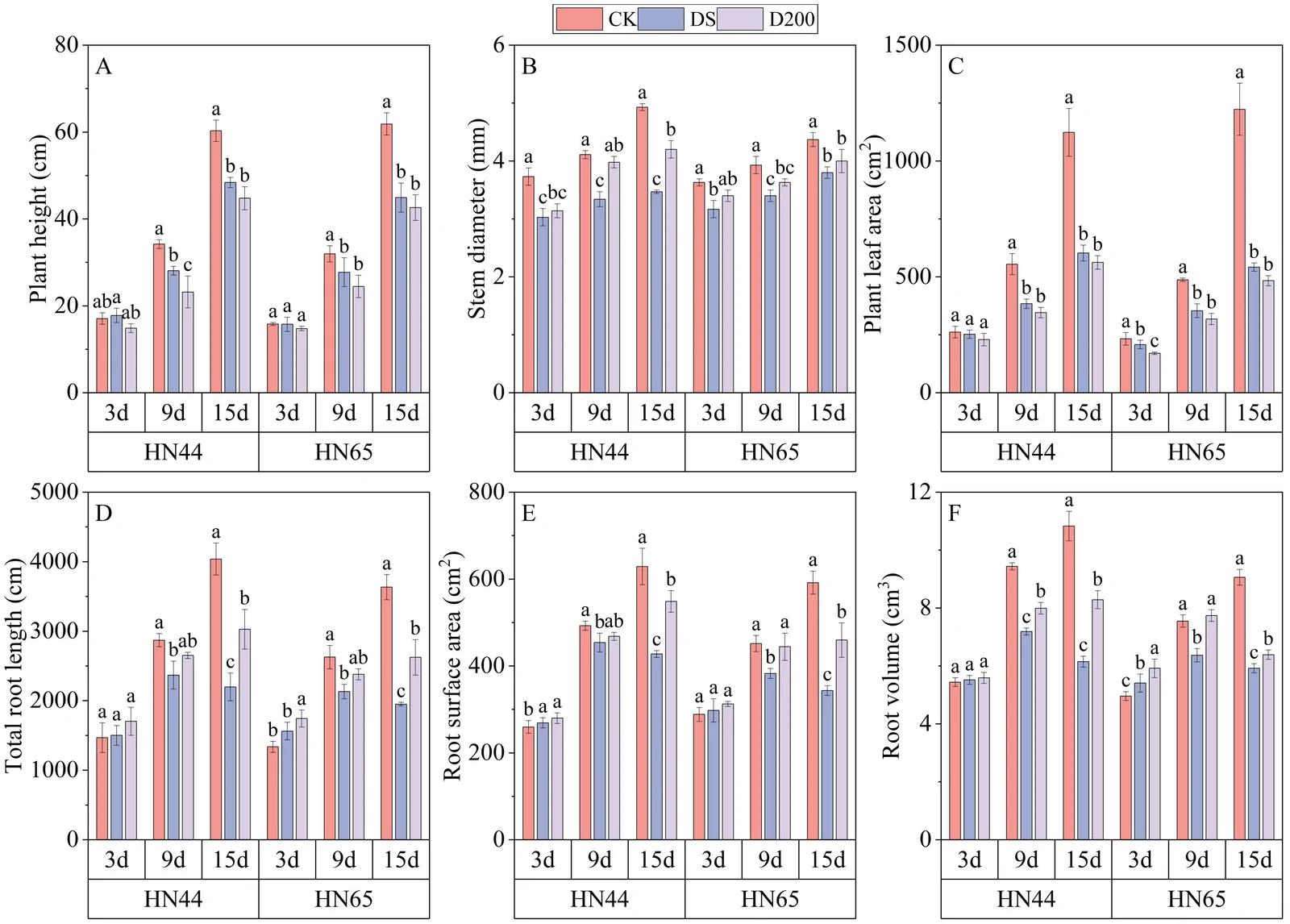
Effects of DPC application on soybean morphology under drought stress. **(A)** Plant height; **(B)** Stem diameter; **(C)** Plant leaf area; **(D)** Total root length; **(E)** Root surface area; and **(F)** Root volume. Data are presented as means ± SD (n = 3 independent biological replicates). Different lowercase letters indicate significant differences among treatments within the same cultivar and sampling time according to Duncan’s multiple range test (P < 0.05). CK, control; DS, drought stress; and D200, drought stress plus 200 mg L^-^¹ DPC.

As shown in **Figure 7**, the effects of drought stress on shoot morphology became more pronounced as stress duration increased. At 3 d, plant height and leaf area differed only slightly among treatments. At 15 d, plant height under DS was 19.72% lower in HN44 and 27.37% lower in HN65 than in CK, whereas leaf area was 46.32% and 55.69% lower, respectively (**Figures 7A, C**). D200 further reduced plant height and leaf area relative to DS. At 15 d, plant height was 7.52% lower in HN44 and 5.16% lower in HN65, and leaf area was 6.76% and 10.77% lower, respectively. Stem diameter increased under D200, particularly in HN44 (**Figure 7B**). At 15 d, stem diameter under D200 was 21.04% greater in HN44 and 5.26% greater in HN65 than under DS.

In contrast to the shoot response, D200 increased root traits under drought stress, with differences becoming more apparent at 9 and 15 d (**Figures 7D–F**). At 15 d, total root length under D200 was 37.75% greater in HN44 and 34.50% greater in HN65 than under DS. Root surface area increased by 28.46% and 33.37%, respectively. The root-volume response differed between cultivars. In HN44, the increase under D200 became greater with stress duration and reached 34.80% at 15 d. In HN65, root volume increased initially and then declined, with a 7.94% increase at 15 d.

### 3.8 Effects of DPC application on soybean biomass under drought stress

**Figure 8:**
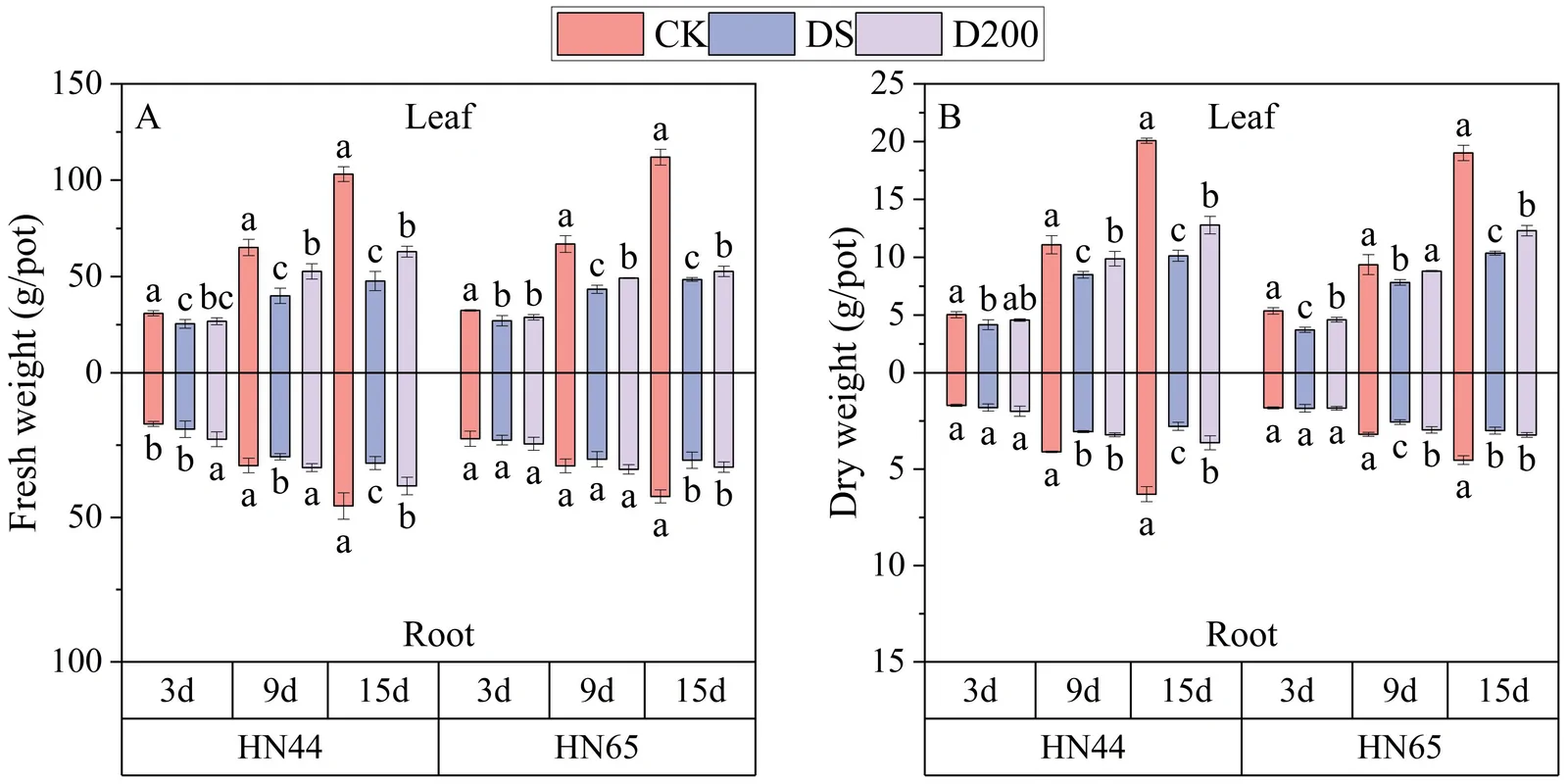
Effects of DPC application on soybean biomass under drought stress. **(A)** Fresh weight; and **(B)** Dry weight. Data are presented as means ± SD (n = 3 independent biological replicates). Different lowercase letters indicate significant differences among treatments within the same cultivar and sampling time according to Duncan’s multiple range test (P < 0.05). CK, control; DS, drought stress; and D200, drought stress plus 200 mg L^-^¹ DPC.

As shown in **Figure 8**, drought stress reduced shoot biomass in both cultivars, and the reduction became greater with stress duration. At 15 d, shoot fresh weight under DS was 53.79% lower in HN44 and 56.72% lower in HN65 than in CK. Shoot dry weight was 49.60% lower in HN44 and 45.58% lower in HN65. Relative to DS, D200 increased shoot fresh weight by 31.92% in HN44 and 8.77% in HN65 at 15 d. Shoot dry weight increased by 26.28% and 18.74%, respectively.

Root biomass showed a different temporal response. At 3 d, root fresh and dry weights under DS were slightly higher than those under CK in both cultivars. In HN44, fresh and dry weights increased by 10.41% and 2.16%, respectively; in HN65, they increased by 6.81% and 1.12%. At 9 and 15 d, root biomass under DS declined. At 15 d, root fresh weight was 32.24% lower in HN44 and 29.37% lower in HN65 than in CK, whereas root dry weight was 55.98% and 33.84% lower, respectively. Relative to DS, D200 increased root fresh and dry weights in HN44 by 25.40% and 30.89%, respectively. The corresponding increases in HN65 were 7.97% and 7.55%.

### 3.9 Effects of DPC application on photosynthetic characteristics and SPAD under drought stress

**Figure 9:**
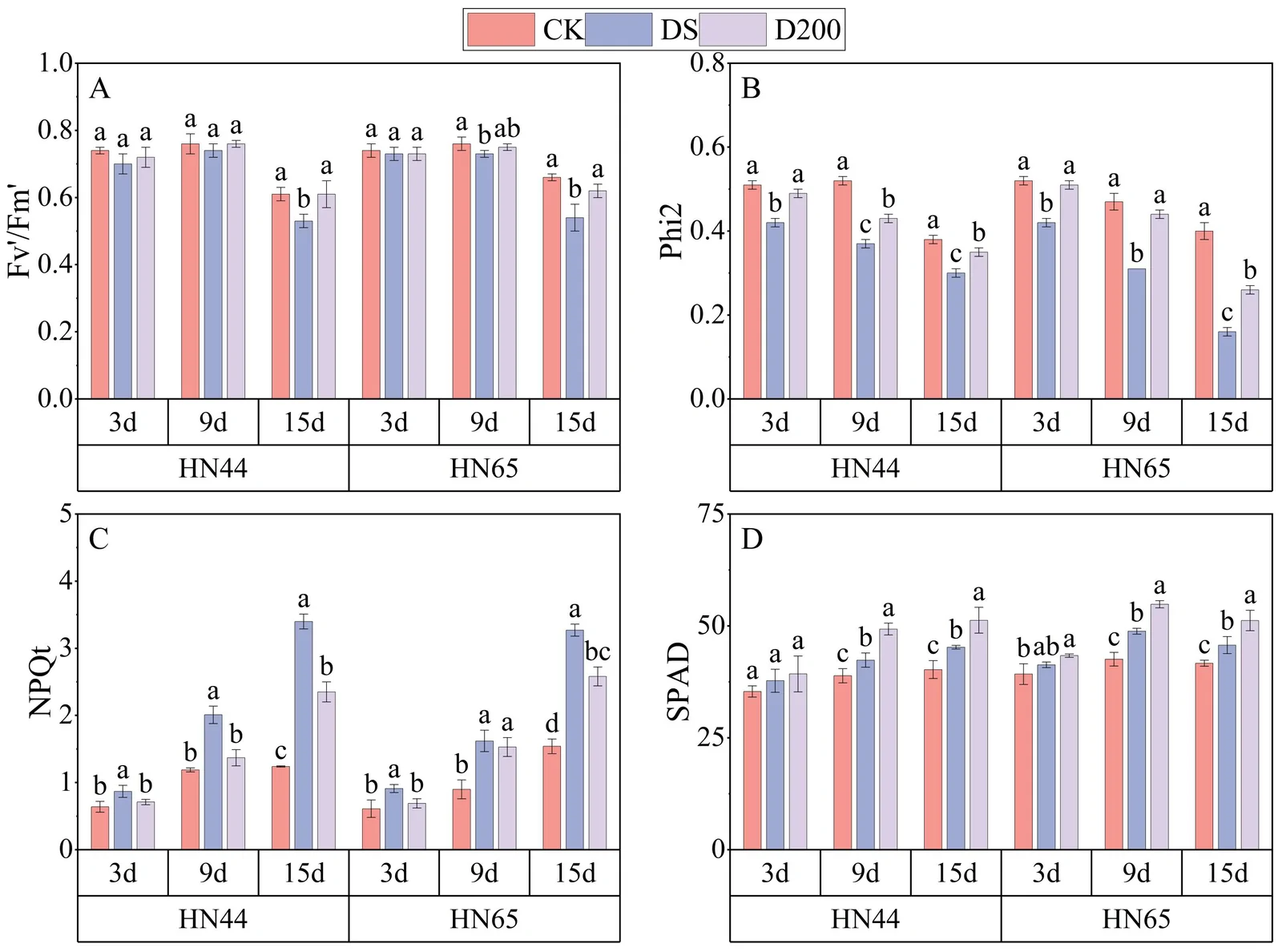
Effects of DPC application on soybean photosynthetic characteristics and SPAD under drought stress. **(A)** Maximum photochemical efficiency of photosystem II under light (Fv′/Fm′); **(B)** Effective quantum yield of photosystem II (Phi2); **(C)** Non-photochemical quenching (NPQt); and **(D)** SPAD value. Data are presented as means ± SD (n = 3 independent biological replicates). Different lowercase letters indicate significant differences among treatments within the same cultivar and sampling time according to Duncan’s multiple range test (P < 0.05). CK, control; DS, drought stress; and D200, drought stress plus 200 mg L^-^¹ DPC.

As shown in **Figure 9**, drought stress altered leaf chlorophyll fluorescence parameters in both cultivars. Fv′/Fm′ and Phi2 declined with stress duration under DS (**Figures 9A, B**). At 15 d, Fv′/Fm′ and Phi2 in HN44 were 13.10% and 21.09% lower, respectively, under DS than under CK. The corresponding reductions in HN65 were 18.19% and 59.75%. D200 increased Fv′/Fm′ and Phi2 relative to DS. At 15 d, Fv′/Fm′ under D200 did not differ significantly from CK in either cultivar. Phi2 remained lower than CK, but the reduction under D200 was 8.62% in HN44 and 33.64% in HN65.

NPQt increased under DS in both cultivars (**Figure 9C**). At 15 d, NPQt was 174.38% higher in HN44 and 112.45% higher in HN65 than under CK. Under D200, the corresponding increases were 89.82% and 67.83%. SPAD values also increased under DS (**Figure 9D**). At 15 d, SPAD increased by 12.43% in HN44 and 9.75% in HN65 relative to CK. Under D200, SPAD was 27.36% higher in HN44 and 22.90% higher in HN65 than under CK.

### 3.10 Effects of DPC application on MDA and H_2_O_2_ in soybean under drought stress

**Figure 10:**
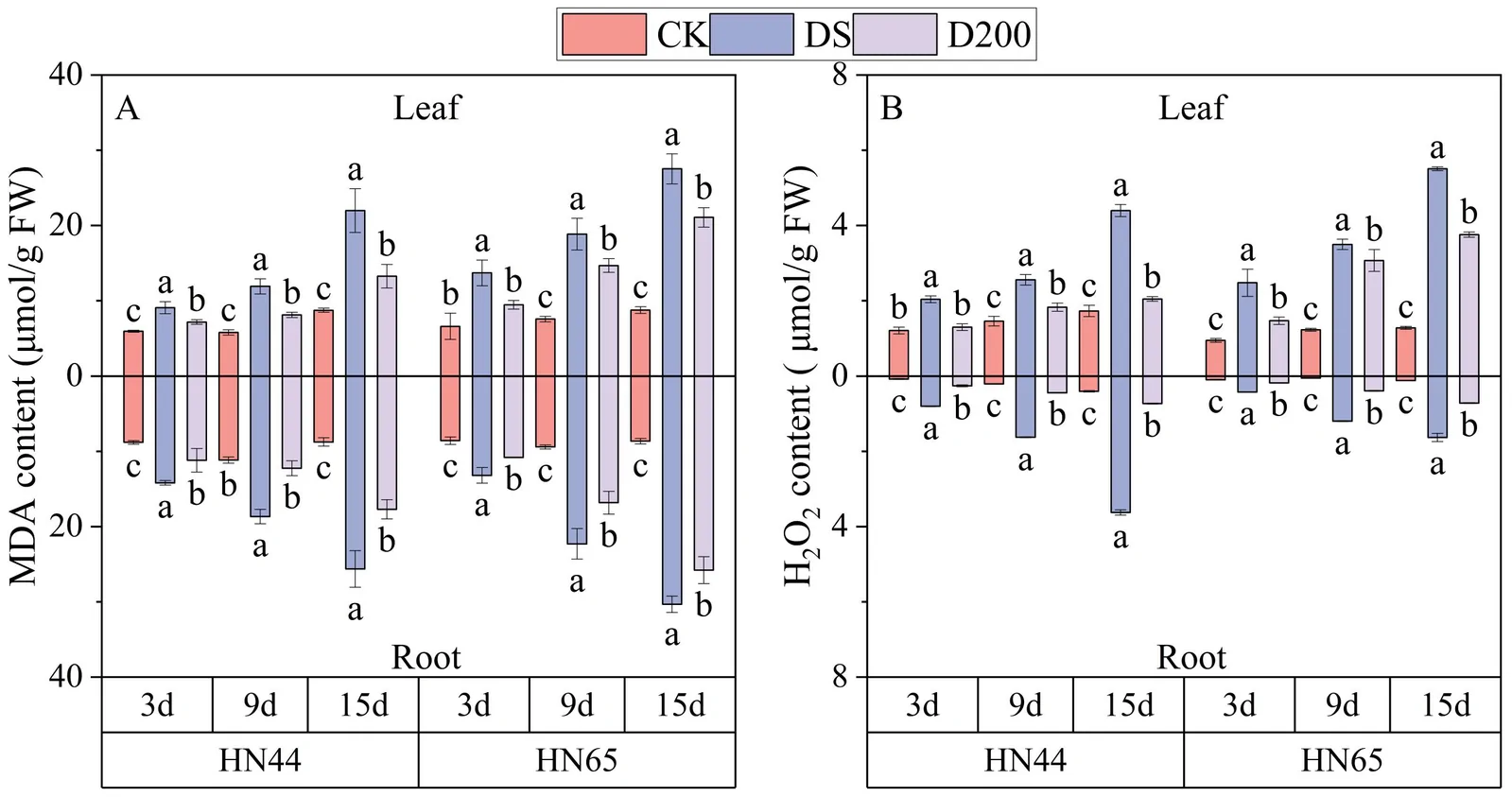
Effects of DPC application on MDA and H_2_O_2_ under drought stress. **(A)** MDA content; **(B)** H_2_O_2_ content. Data are presented as means ± SD (n = 3 independent biological replicates). Different lowercase letters indicate significant differences among treatments within the same cultivar and sampling time according to Duncan’s multiple range test (P < 0.05). CK, control; DS, drought stress; and D200, drought stress plus 200 mg L^-^¹ DPC.

As shown in **Figure 10A**, MDA contents in leaves and roots increased with drought duration in both cultivars. At 15 d, leaf MDA content under DS was 151.15% higher in HN44 and 214.27% higher in HN65 than under CK. Root MDA content increased by 192.47% and 250.76%, respectively. Relative to DS, D200 reduced leaf MDA content by 39.68% in HN44 and 23.43% in HN65. In roots, D200 reduced MDA content by 30.87% in HN44 and 14.91% in HN65.

H_2_O_2_ content showed a similar pattern (**Figure 10B**). At 15 d, leaf H_2_O_2_ content under DS increased by 154.34% in HN44 and 330.47% in HN65 relative to CK. Root H_2_O_2_ content increased by 791.69% and 1244.19%, respectively. Relative to DS, D200 reduced leaf H_2_O_2_ content by 53.41% in HN44 and 31.76% in HN65. Root H_2_O_2_ content decreased by 79.84% in HN44 and 55.82% in HN65.

### 3.11 Effects of DPC application on antioxidant enzymes and osmotic regulation in soybean under drought stress

**Figure 11:**
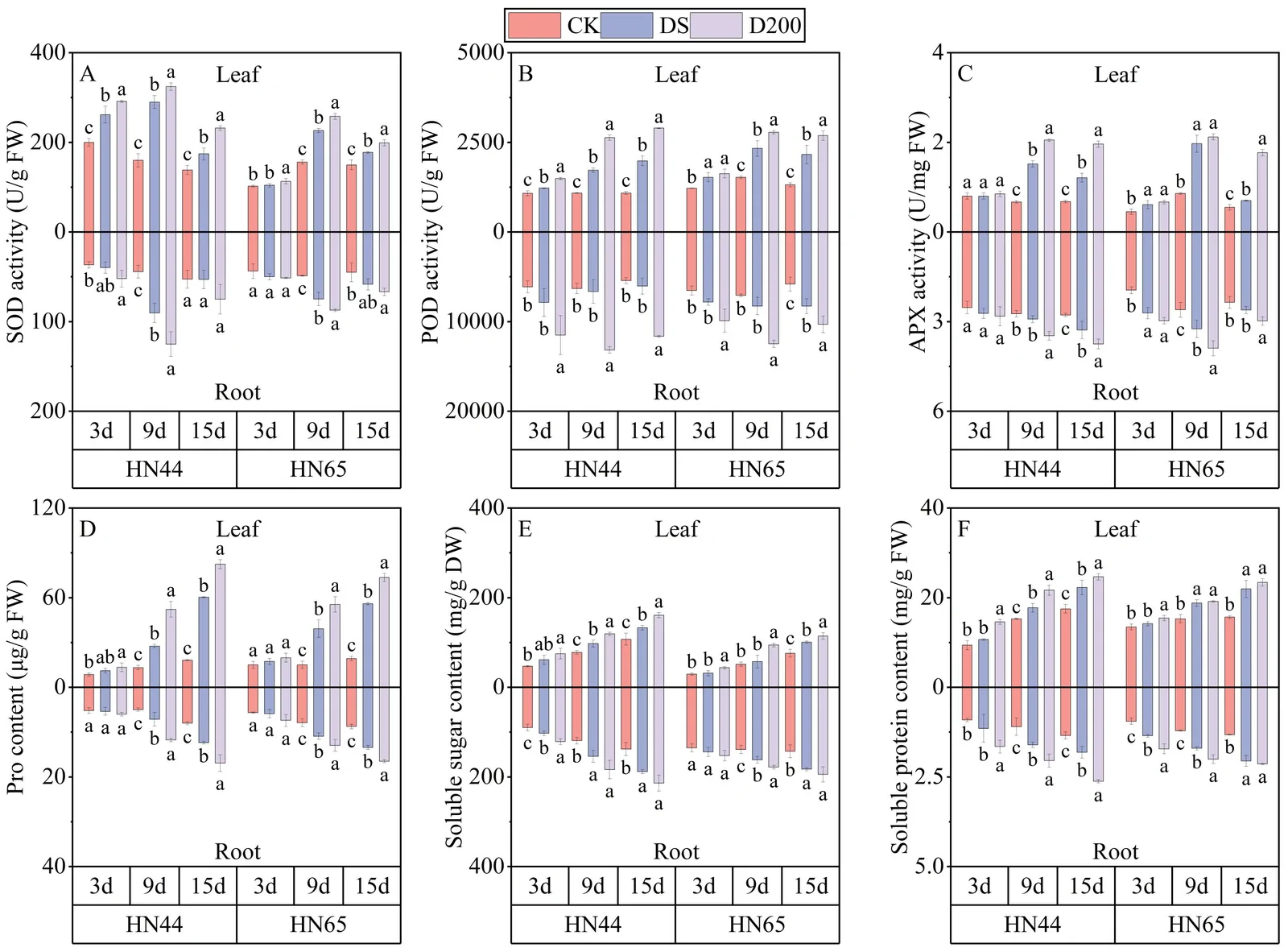
Effects of DPC application on antioxidant enzymes and osmotic regulation under drought stress. **(A)** SOD activity; **(B)** POD activity; **(C)** APX activity; **(D)** Proline content; **(E)** Soluble sugar content; and **(F)** Soluble protein content in leaves and roots. Data are presented as means ± SD (n = 3 independent biological replicates). Different lowercase letters indicate significant differences among treatments within the same cultivar and sampling time according to Duncan’s multiple range test (P < 0.05). CK, control; DS, drought stress; and D200, drought stress plus 200 mg L^-^¹ DPC.

As shown in **Figures 11A–C**, SOD, POD, and APX activities increased in leaves and roots of both cultivars during drought stress. D200 further increased enzyme activities, which generally increased and then decreased during the stress period, with most values peaking at 9 d. At 9 d, D200 increased SOD and POD activities in leaves and roots of both cultivars relative to DS. The largest increase was observed for POD activity in HN44 roots, which was 98.49% greater than under DS. The APX response differed between cultivars. In HN44, the largest D200-associated increases in root and leaf APX activity occurred at 15 d, whereas in HN65 they occurred at 9 d.

Pro, soluble sugar (SS), and soluble protein (SP) contents in leaves and roots increased with drought duration and were greatest at 15 d (**Figures 11D–F**). At 15 d, D200 increased Pro, SS, and SP contents in leaves by 10.73%–36.64% in HN44 and by 6.59%–31.20% in HN65 relative to DS. In roots, the corresponding increases were 13.57%–44.75% in HN44 and 3.90%–22.90% in HN65.

### 3.12 Effects of DPC application on phenylpropanoid metabolism in soybean under drought stress

**Figure 12:**
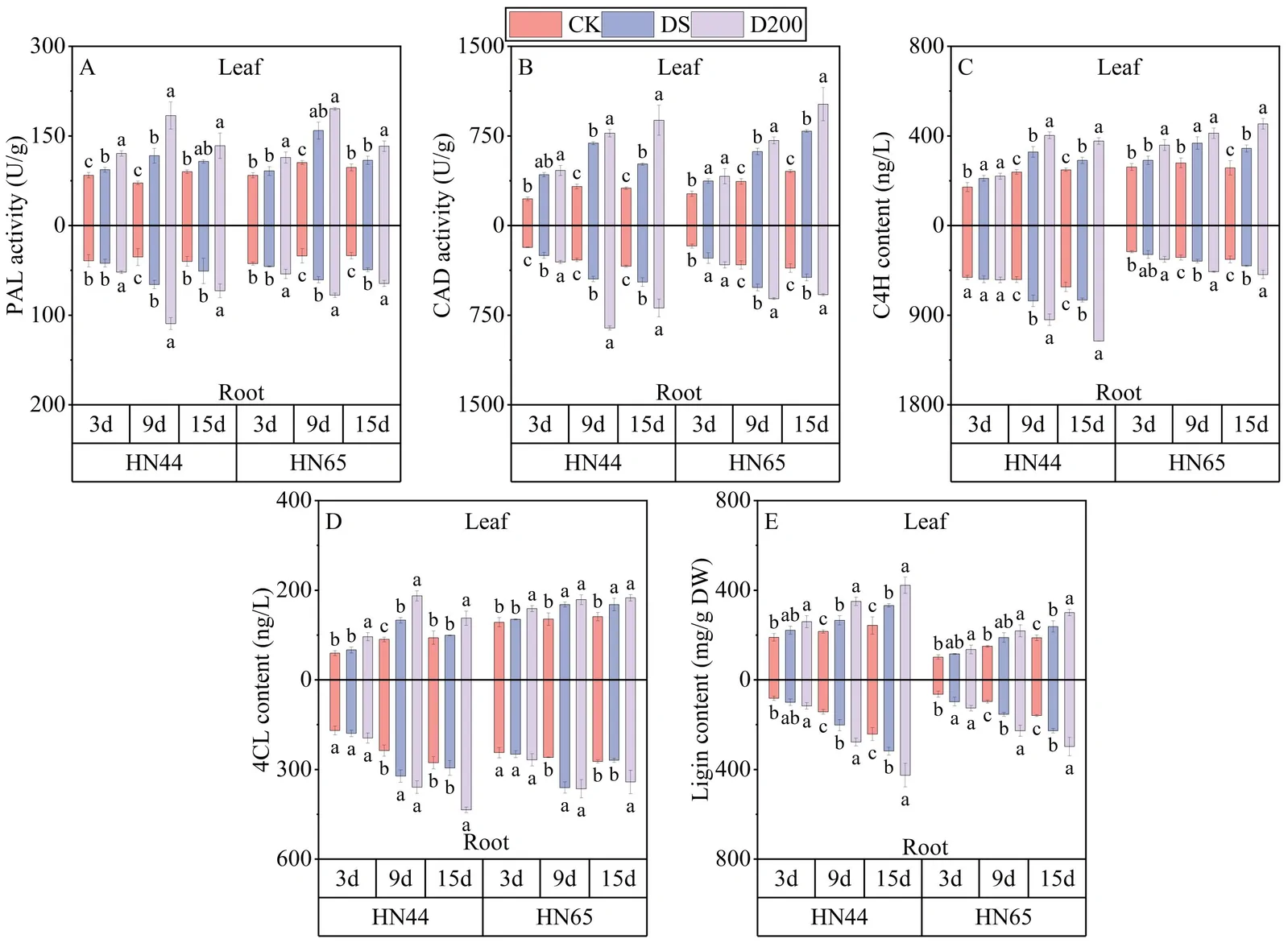
Effects of DPC application on phenylpropanoid metabolism under drought stress. **(A)** PAL activity; **(B)** C4H activity; **(C)** CAD activity; **(D)** 4CL activity; and **(E)** Lignin content in leaves and roots. Data are presented as means ± SD (n = 3 independent biological replicates). Different lowercase letters indicate significant differences among treatments within the same cultivar and sampling time according to Duncan’s multiple range test (P < 0.05). CK, control; DS, drought stress; and D200, drought stress plus 200 mg L^-^¹ DPC.

As shown in **Figure 12**, PAL, 4CL, C4H, and CAD activities increased under drought stress, although their temporal patterns differed. PAL and 4CL activities in leaves and roots generally increased and then declined, with most values peaking at 9 d (**Figures 12A, D**). At 9 d, 4CL activity increased by 47.69% in HN44 leaves and 24.05% in HN65 leaves relative to CK, and by 35.86% and 38.93%, respectively, in roots. PAL activity increased by 63.90% and 50.53% in leaves and by 87.58% and 78.74% in roots. Relative to DS, D200 increased PAL activity by 57.63% and 23.06% in HN44 and HN65 leaves, respectively, and by 66.35% and 28.49% in roots. D200 also increased 4CL activity by 40.74% and 6.34% in leaves and by 11.61% and 1.21% in roots, respectively.

C4H and CAD activities showed different temporal patterns between cultivars (**Figures 12B, C**). In HN44, leaf and root activities increased and then declined, peaking at 9 d. At this time, C4H and CAD activities in HN44 leaves were 37.46% and 110.92% higher, respectively, than under CK; the corresponding increases in roots were 39.59% and 55.59%. In HN65, except for root CAD activity, which peaked at 3 d, the other activities generally increased with stress duration and peaked at 15 d. At 15 d, C4H and CAD activities in HN65 leaves were 33.67% and 73.90% higher, respectively, than under CK, and root activities were 18.79% and 21.89% higher. Relative to DS, D200 increased leaf C4H activity by 29.64% in HN44 and 31.81% in HN65 at 15 d, and increased CAD activity by 71.35% and 28.51%, respectively. In roots, D200 increased C4H activity by 55.05% and 21.52% and CAD activity by 45.76% and 33.33%, respectively.

Lignin content showed a temporal pattern broadly consistent with the enzyme activities (**Figure 12E**). At 15 d, lignin content under DS was 36.75% higher in HN44 leaves and 26.55% higher in HN65 leaves than under CK. Root lignin content increased by 31.23% and 42.31%, respectively. Relative to DS, D200 increased leaf lignin content by 27.02% in HN44 and 26.26% in HN65, and root lignin content by 33.94% and 28.54%, respectively.

### 3.13 Effects of DPC application on auxin metabolism in soybean under drought stress

**Figure 13:**
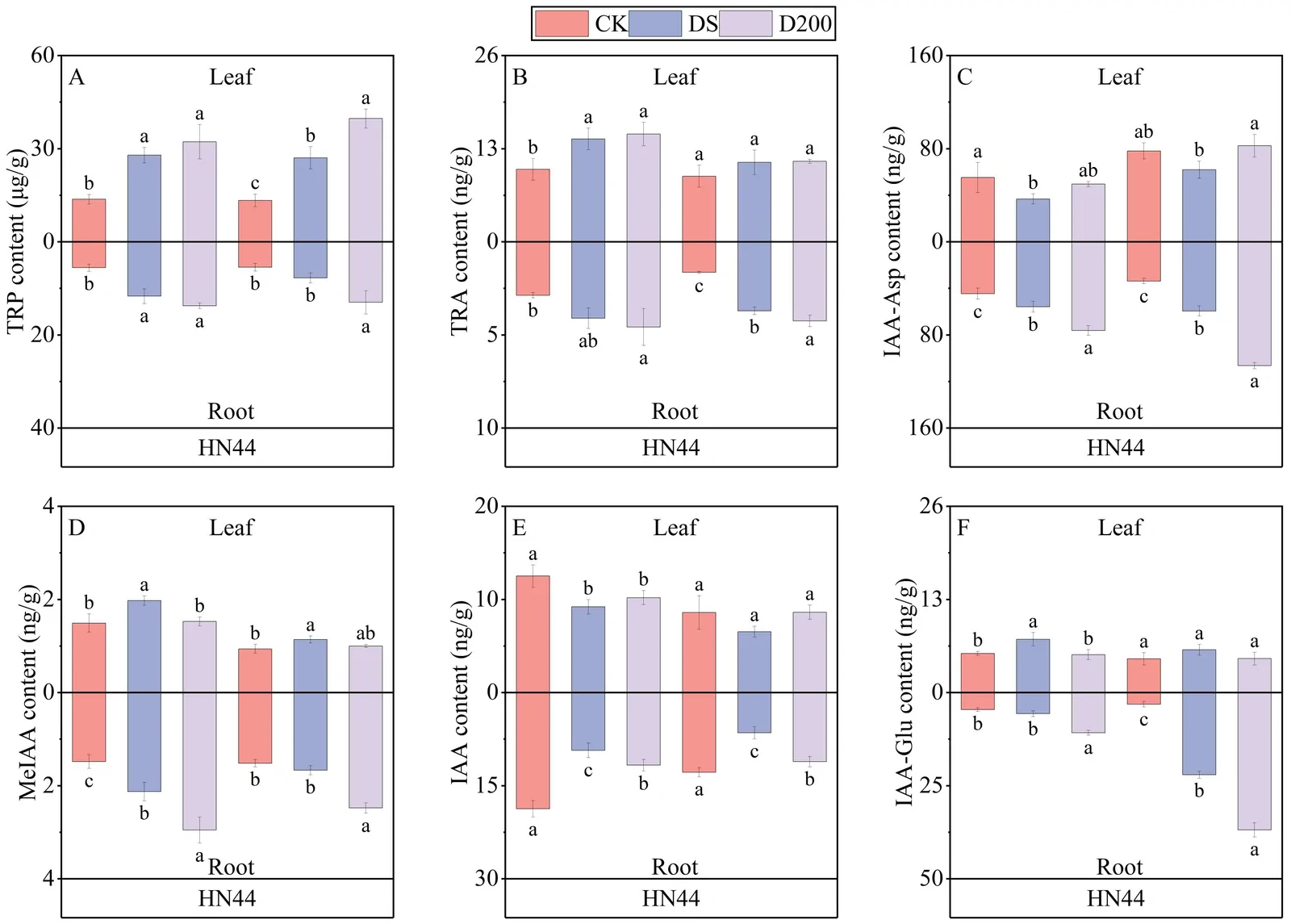
Effects of DPC application on auxin and auxin metabolite contents in soybean under drought stress. **(A)** TRP; **(B)** TRA; **(C)** IAA-Asp; **(D)** MeIAA; **(E)** IAA; and **(F)** IAA-Glu contents in leaves and roots. Data are presented as means ± SD (n = 3 independent biological replicates). Different lowercase letters indicate significant differences among treatments within the same cultivar according to Duncan’s multiple range test (P < 0.05). CK, control; DS, drought stress; and D200, drought stress plus 200 mg L^-^¹ DPC.

Based on changes in morphological and physiological indicators from 3 to 15 d, the effects of drought stress and DPC treatment had not yet fully emerged at 3 d, whereas the markedly elevated accumulation of H_2_O_2_ and MDA at 15 d indicated that the plants had entered a stage of severe oxidative damage. In addition, the activities of antioxidant enzymes and key enzymes involved in phenylpropanoid metabolism generally peaked at 9 d of stress. Therefore, 9 d after treatment was selected as the sampling time for the determination of hormone levels in leaves and roots. Drought stress significantly altered auxin metabolic pathways in soybean leaves and roots, whereas DPC application effectively contributed to the maintenance of auxin homeostasis in plants (**Figure 13**).

Drought stress and DPC treatment significantly altered the balance between auxin synthesis and metabolism in soybean. With respect to auxin synthesis, DS significantly promoted the accumulation of TRP and TRA (**Figures 13A, B**), and D200 further enhanced their accumulation, indicating that DPC may strengthen the upstream precursor supply for auxin synthesis under drought stress. However, despite the increased levels of these precursors, IAA contents in the leaves and roots of both cultivars decreased significantly (**Figure 13E**), with reductions of approximately 50% in roots. DPC application significantly alleviated the drought-induced inhibition of IAA synthesis. Under D200, IAA contents in the leaves and roots of both cultivars recovered to varying degrees relative to those under DS. Specifically, leaf IAA contents in HN44 and HN65 increased by 10.69% and 32.07%, respectively, whereas root IAA contents increased by 25.80% and 71.76%, respectively, compared with those under DS.

In addition, drought stress significantly altered auxin inactivation and storage pathways. Under DS, MeIAA and IAA-Glu contents generally increased in soybean plants (**Figures 13D, F**). Compared with CK, MeIAA contents in the leaves and roots of HN44 increased by 32.40% and 43.46%, respectively, whereas those in HN65 increased by 21.40% and 9.89%, respectively. Similarly, IAA-Glu contents in the leaves and roots of HN44 increased by 35.94% and 25.19%, respectively, while those in HN65 increased by 26.89% and 609.01%, respectively. DPC significantly modified auxin inactivation and storage pathways in a tissue-specific manner (**Figures 13C, D, F**). In leaves, D200 inhibited the excessive accumulation of MeIAA and IAA-Glu, resulting in lower contents than those observed under DS. In roots, however, D200 significantly increased the levels of both compounds, particularly in HN65 roots, where the increases reached 48.65% and 67.08%, respectively. At the same time, D200 further increased the content of conjugated IAA-Asp in both leaves and roots, with a more pronounced increase in roots. Compared with DS, root IAA-Asp contents increased by 36.62% and 79.02% in HN44 and HN65, respectively.

### 3.14 Effects of DPC application on ABA, jasmonates, and cytokinins in soybean under drought stress

**Figure 14:**
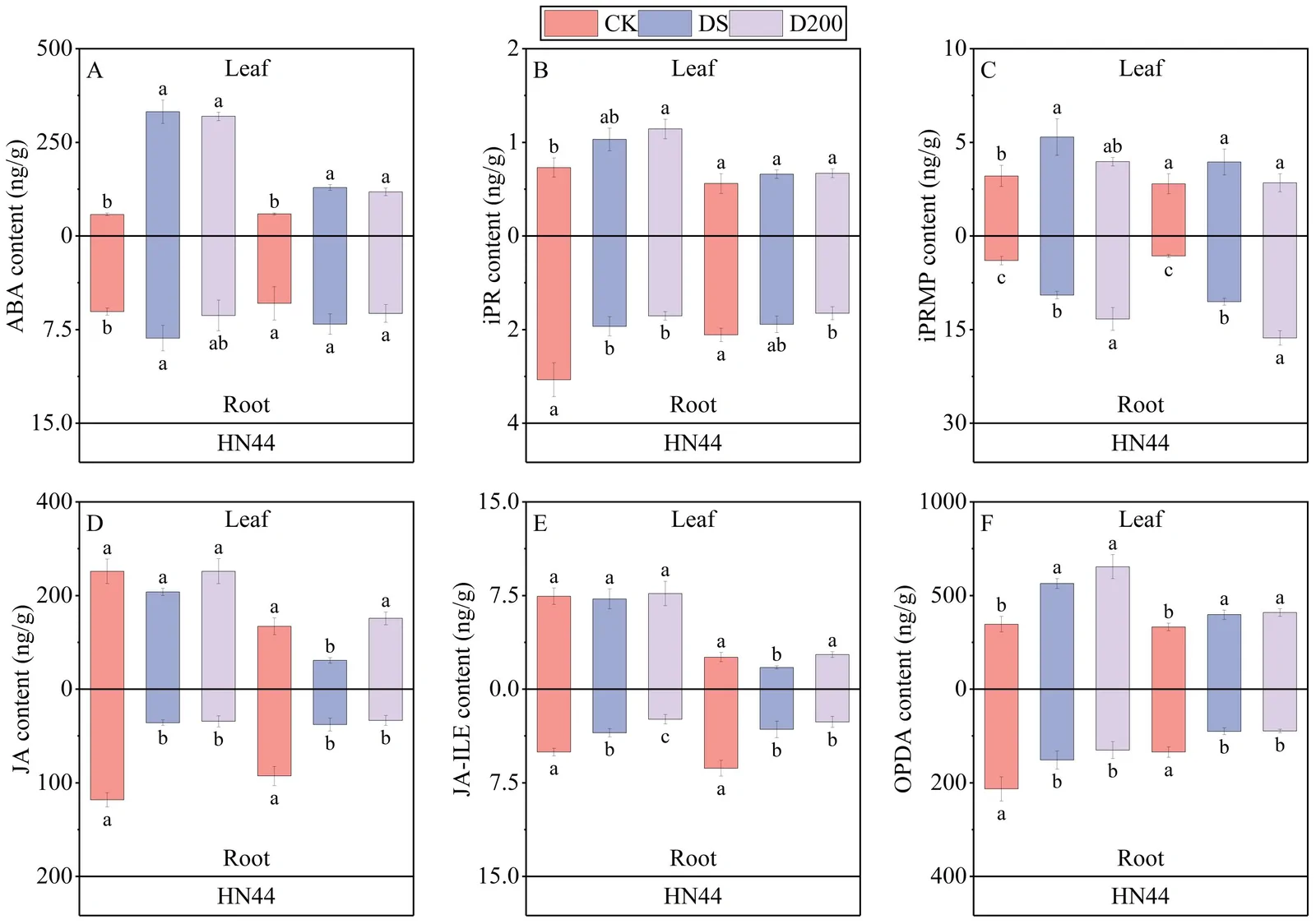
Effects of DPC application on ABA, jasmonates, and cytokinins in soybean under drought stress. **(A)** ABA; **(B)** iPR; **(C)** iPRMP; **(D)** JA; **(E)** JA-Ile; and **(F)** OPDA contents in leaves and roots. Data are presented as means ± SD (n = 3 independent biological replicates). Different lowercase letters indicate significant differences among treatments within the same cultivar according to Duncan’s multiple range test (P < 0.05). CK, control; DS, drought stress; and D200, drought stress plus 200 mg L^-^¹ DPC.

Abscisic acid (ABA), a key hormone involved in drought responses, increased significantly under DS. Compared with CK, DS increased leaf ABA contents by 477.42% in HN44 and 119.28% in HN65, and root ABA contents by 35.36% and 30.64%, respectively (**Figure 14A**). DPC application significantly reduced ABA levels under drought stress. Under D200, leaf ABA contents decreased by 3.66% in HN44 and 8.96% in HN65 relative to DS, whereas root ABA contents decreased by 22.20% and 12.13%, respectively. For cytokinins, drought stress increased iPR and iPRMP contents in leaves. Following DPC application, leaf iPR contents increased further, by 10.92% in HN44 and 1.52% in HN65 under D200 relative to DS. In contrast, leaf iPRMP contents decreased significantly, by 24.85% and 28.21%, respectively. The response pattern in roots was opposite to that observed in leaves. Drought stress significantly reduced root iPR contents, and DPC treatment further decreased them. Under D200, root iPR contents decreased by 11.54% in HN44 and 12.58% in HN65 relative to DS, whereas root iPRMP contents increased by 40.36% and 55.06%, respectively (**Figures 14B, C**).

DPC treatment induced distinct jasmonate responses in leaves and roots. In leaves, drought stress reduced JA contents, whereas DPC treatment partially alleviated this reduction. Under D200, leaf JA contents increased by 21.42% in HN44 and 146.23% in HN65 relative to DS (**Figure 14D**). In addition, the active conjugate JA-Ile and its precursor OPDA increased under DS and increased further following DPC treatment. Compared with DS, D200 increased JA-Ile contents by 5.95% in HN44 and 58.97% in HN65, and increased OPDA contents by 15.85% and 2.89%, respectively. In contrast, drought stress reduced root JA, JA-Ile, and OPDA contents. Following DPC treatment, no significant differences were observed for most root jasmonate-related indicators relative to DS, except for JA-Ile in HN44 (**Figures 14E, F**).

### 3.15 Analysis of variance

Multifactor analysis of variance (ANOVA) showed that drought stress and DPC treatment significantly affected physiological, morphological, and endogenous hormone traits in different soybean organs (**Supplementary Tables 3**-**5**). For leaf physiological and morphological traits, the main effects of treatment and sampling time were extremely significant for all measured variables, indicating that drought stress and exogenous DPC application substantially influenced leaf morphology and physiological status. In addition, the treatment × time interaction was extremely significant for most traits, suggesting that leaf responses to DPC treatment varied over time. Furthermore, the cultivar × treatment × time interaction was significant or extremely significant for antioxidant traits, including H_2_O_2_ and APX, as well as morphological traits, including stem diameter and leaf area.

For root traits, treatment and sampling time also had extremely significant effects on all physiological and morphological variables. In particular, the cultivar main effect showed high F values for root H_2_O_2_ content and C4H activity, indicating substantial differences between the two soybean cultivars in root oxidative damage and secondary metabolism. Regarding interaction effects, the cultivar × treatment × time interaction was extremely significant (P < 0.001) for root H_2_O_2_, MDA, C4H, CAD, and root volume.

ANOVA of endogenous hormones further demonstrated the effects of DPC treatment on hormone contents. Treatment had extremely significant effects on the contents of most hormones in leaves and roots, particularly auxins and jasmonates. In leaves, the cultivar × treatment interaction was extremely significant for ABA and OPDA. In roots, this interaction was significant for several growth- and stress-related hormones, including IAA, IAA-Asp, IAA-Glu, iPR, and JA. These results indicate that DPC treatment was associated with changes in endogenous hormone contents in soybean under drought stress and that these responses differed between the two cultivars.

### 3.16 Integrated physiological responses of DPC in alleviating drought stress in soybean

**Figure 15:**
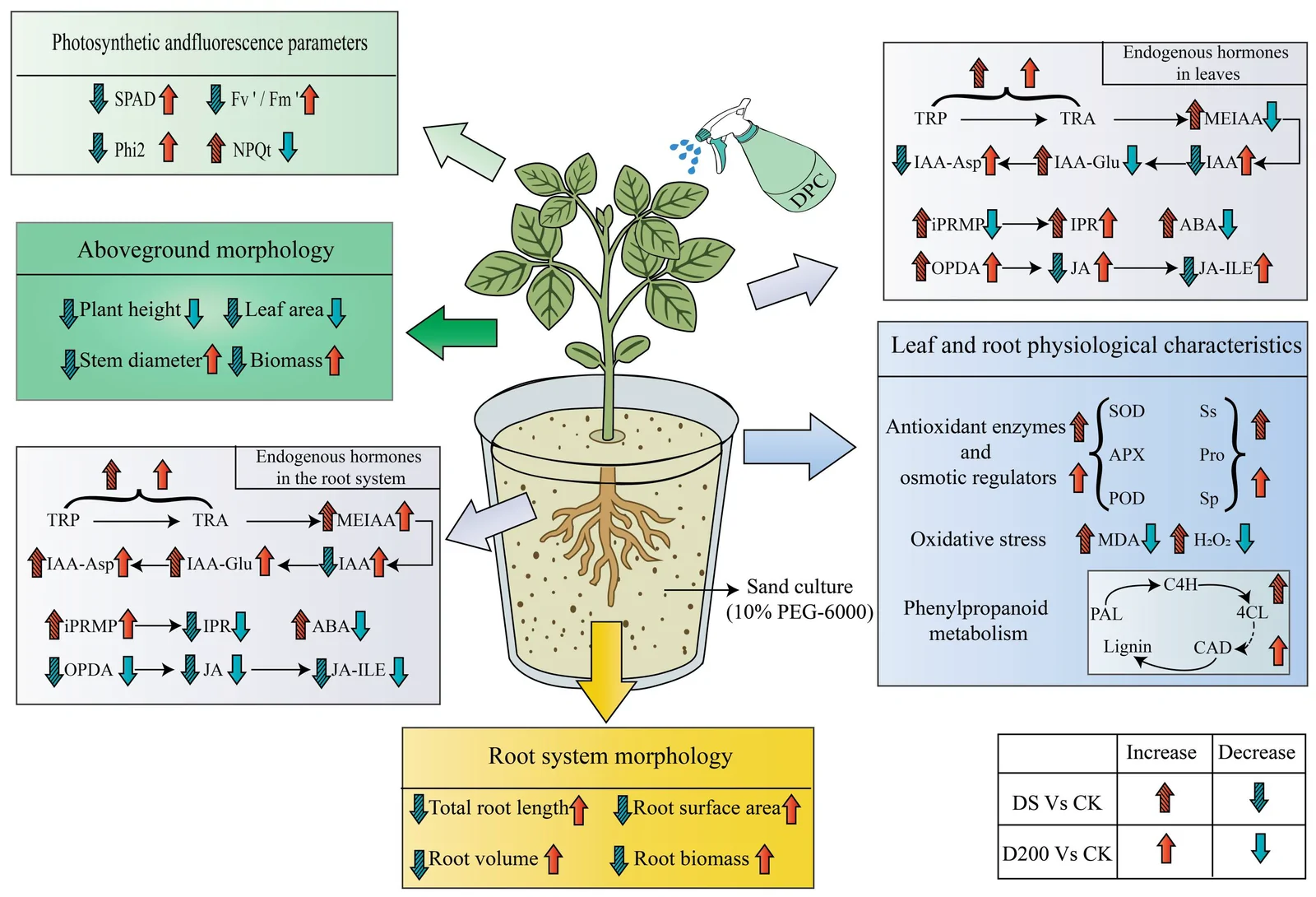
Conceptual model of morphological and physiological responses associated with DPC treatment in soybean under drought stress. The relationships shown are proposed based on the responses observed in the present study.

As shown in **Figure 15**, the integrated analysis indicated that DPC treatment was associated with coordinated morphological and physiological responses in soybean under drought stress. DPC moderately inhibited plant height and leaf-area expansion, while promoting increases in total root length, root surface area, root volume, and stem diameter. These changes may contribute to a more favorable balance between water consumption and water acquisition. In terms of photosynthesis, DPC treatment increased leaf SPAD values and chlorophyll fluorescence parameters under drought stress while reducing NPQt, indicating improved maintenance of photosynthetic performance. In leaves and roots, DPC treatment was also associated with enhanced antioxidant enzyme activities and osmotic-regulation capacity, accompanied by lower H_2_O_2_ and MDA contents. In addition, DPC treatment increased the activities of key phenylpropanoid-metabolism-related enzymes and lignin accumulation, which may contribute to enhanced structural stability under drought stress.

The endogenous hormone profiles showed distinct responses between roots and leaves following DPC treatment. In leaves, DPC treatment was associated with lower ABA contents and higher JA and IAA contents under drought stress. In roots, DPC-treated plants showed changes in TRP, TRA, MeIAA, IAA, and related auxin metabolites, which were accompanied by improved root morphological traits. ABA- and JA-related compounds also showed different response patterns between roots and leaves. These results indicate that hormone-related metabolites in roots and leaves exhibited distinct response patterns to DPC treatment, which may be associated with differential aboveground and belowground responses of soybean under drought stress.

## 4 Discussion

Plant growth regulators play important roles in regulating crop canopy architecture and improving yield (Rademacher, 2015). In the present study, exogenous DPC application significantly reduced soybean plant height and internode length while promoting stem thickening. These effects reflect the ability of DPC, a gibberellin (GA) biosynthesis inhibitor, to inhibit longitudinal cell division and elongation (Shi et al., 2022). However, internode sensitivity to DPC differed between cultivars. HN44 was mainly responsive at nodes 6–14, whereas HN65 was mainly responsive at nodes 5–10. This difference may be related to cultivar-specific differences in the period of rapid internode elongation at the same developmental stage. DPC-mediated remodeling of plant architecture not only improved canopy ventilation and light penetration at the population level, thereby increasing light capture by lower leaves, but also shortened the distance for photosynthate transport to reproductive organs, providing a morphological basis for assimilate allocation to sink organs (Wu et al., 2023). In this study, DPC treatment significantly increased pod number per plant, particularly the numbers of two-seed and three-seed pods, while reducing ineffective pod number. PCA further confirmed that soybean yield was highly and positively correlated with seed number per plant and the numbers of two-seed and three-seed pods, whereas its correlation with 100-seed weight was relatively weak. These results indicate that the yield-increasing effect of DPC was achieved not by increasing seed weight, but by optimizing pod-setting structure, increasing the proportion of effective pods, and increasing seed number per plant (Wang et al., 2025). However, when the DPC concentration reached 500 mg L^-^¹, total pod number and yield decreased to varying degrees in both cultivars. This result suggests that excessive DPC application excessively inhibited vegetative growth and restricted photosynthetic area, thereby failing to meet the energy and carbohydrate demands of reproductive growth (Tung et al., 2018).

The yield response to DPC differed between the two cultivars under the field conditions of the present study. HN44 showed the greatest yield increase under D200 (29.44%), whereas the highest numerical increase in HN65 was observed under D275 (12.99%). However, the yield of HN65 under D200 did not differ significantly from that under D275. These dose-response patterns suggest that the two cultivars differed in their sensitivity to DPC-mediated yield regulation.

Given that DPC-induced yield improvement in the present study was mainly associated with enhanced effective pod formation and seed number rather than increased 100-seed weight, the stronger response of HN44 to D200 may indicate that this cultivar coordinated DPC-induced architectural adjustment and reproductive development more effectively, thereby supporting effective pod formation and seed production. By contrast, the relatively smaller yield response of HN65 may suggest less favorable coordination between DPC-induced architectural adjustment and yield formation at the same dose. Nevertheless, this interpretation should be treated with caution because pod abortion, canopy photosynthesis, assimilate partitioning, and source–sink relationships were not directly measured in the field experiment. Previous studies have also shown that soybean cultivars may differ in their growth and yield responses to DPC concentration under non-drought conditions (Wang et al., 2025). It should also be noted that the observed difference between HN44 and HN65 mainly reflects differential DPC-mediated regulation of yield formation under the natural field conditions of the present study. Because no drought treatment was imposed and soil water dynamics were not monitored, the field experiment was used primarily to evaluate the effects of DPC on plant architecture and yield formation. Drought-related physiological responses were examined separately in the PEG-induced simulated-drought experiment. Accordingly, the selection of D200 for the subsequent experiment was supported by its comparable yield response to D275 in HN65 and its superior yield response in HN44.

When crops experience drought stress, they typically adjust shoot and root morphology to balance water loss through transpiration with water uptake (Dao et al., 2025). In the present study, drought stress significantly inhibited soybean plant height and leaf area, and DPC application further intensified these effects. Moderate inhibition of shoot growth can reduce the transpiring area, delay drought-induced wilting, and reduce unnecessary nutrient consumption (Wahab et al., 2022). In addition, DPC promoted stem thickening. Increased stem diameter can improve mechanical support and reduce lodging risk, and it may also be accompanied by changes in vascular-bundle tissues that provide structural support for water and nutrient transport under drought stress (Xinghong et al., 1999; Kamran et al., 2018). In contrast to its inhibitory effects on shoots, DPC promoted increases in total root length, root surface area, and root volume during the middle and late stages of drought stress. This regulatory pattern, characterized by inhibited shoot growth and promoted root growth, directly altered assimilate allocation within the plant. In the present study, the recovery of root fresh and dry weights after DPC treatment was significantly greater than that of shoot biomass, confirming that an appropriate DPC concentration can mediate the preferential transport of photosynthates to belowground organs, support root architecture formation, and thereby enhance root water-interception and uptake capacity (Song et al., 2023). In addition, DPC-mediated regulation of morphology and biomass differed between soybean cultivars with contrasting drought tolerance. Under DPC treatment, the root-growth-promoting effect in HN44 continued to strengthen during the late stage of stress, and its biomass recovery was significantly greater than that of HN65. The stronger root response of HN44 during the later stage of stress may reflect a greater capacity to maintain or increase assimilate allocation to belowground organs under prolonged osmotic stress. Sustained improvements in root biomass and root morphology may enhance the capacity for water exploration and uptake (García‐Rodríguez et al., 2024; Zhang et al., 2025), whereas relatively restricted shoot growth may help reduce transpirational water loss. In contrast, the weaker recovery of root biomass in HN65 suggests that its capacity for growth adjustment under prolonged osmotic stress may be relatively limited.

Drought-induced photosystem dysfunction and ROS accumulation are key factors limiting plant carbon assimilation and causing membrane lipid peroxidation (Qiao et al., 2024; Gao et al., 2025). In this study, Fv′/Fm′ and Phi2 in soybean leaves decreased with increasing drought duration, accompanied by a significant increase in NPQt. These changes indicate that drought caused photoinhibition of PSII reaction centers and forced plants to enhance heat dissipation to consume excess light energy, thereby protecting the photosynthetic apparatus from irreversible oxidative damage (Guo et al., 2018; Suzuki et al., 2021). However, DPC application significantly increased leaf SPAD values, stabilized PSII activity, and reduced NPQt. These findings suggest that the photoprotective effect of DPC did not depend on the activation of heat dissipation, but rather on promoting photosynthetic pigment synthesis and stabilizing PSII reaction centers to maintain efficient electron transport. With respect to physiological homeostasis, SOD, as the first line of defense against ROS, catalyzes the conversion of superoxide anions (O_2_·^-^) into H_2_O_2_, which is subsequently reduced to H_2_O by POD and APX. In addition, the accumulation of osmotic-regulatory substances helps maintain cellular turgor (Singh et al., 2015; Saibi and Brini, 2018; Sharma et al., 2022). In this study, DPC coordinately upregulated SOD, POD, and APX activities in roots and leaves and promoted the accumulation of osmotic-regulatory substances, including Pro, SS, and SP. This coordinated enhancement of antioxidant defense and osmotic regulation significantly inhibited the excessive accumulation of H_2_O_2_ and MDA and alleviated membrane lipid peroxidation damage.

Phenylpropanoid metabolism, a core pathway of plant secondary metabolism, plays an important role in plant adaptation to abiotic stress (Ninkuu et al., 2025). PAL, C4H, and 4CL constitute the core phenylpropanoid pathway, and their catalytic intermediates are ultimately polymerized into lignin through the action of CAD (Dong and Lin, 2021). In this study, DPC treatment significantly increased the activities of key enzymes involved in phenylpropanoid metabolism in soybean leaves and roots under drought stress, thereby promoting lignin synthesis and accumulation. Moreover, lignin accumulation differed between leaves and roots. During the early stage of drought stress, DPC application rapidly promoted lignin accumulation in leaves, which may help increase mesophyll cell-wall rigidity, prevent physical cell collapse caused by water loss, and maintain basic photosynthetic structure (Gall et al., 2015). During the later stage of stress, the regulatory focus of DPC gradually shifted to roots. At this stage, negative pressure inside root xylem vessels increased markedly, making the vessels highly susceptible to deformation, cavitation, or embolism (Schuldt et al., 2025). DPC treatment was associated with increased lignin accumulation in roots during the later stage of drought stress. This response may contribute to cell-wall reinforcement and structural stability in root tissues under prolonged stress. In addition, phenylpropanoid-metabolic responses to DPC treatment differed between the two cultivars. During prolonged osmotic stress, DPC treatment generally exerted a more pronounced promotive effect on the activities of several key phenylpropanoid-metabolism-related enzymes in HN44, which was consistent with its stronger root growth and biomass recovery during the later stage of stress. Increased activities of phenylpropanoid-metabolism-related enzymes and greater lignin accumulation may enhance cell-wall structural stability, thereby helping to maintain tissue integrity under osmotic stress. By comparison, the responses of these enzyme activities in HN65 were relatively weaker or less sustained, which may be associated with its more limited root recovery during the later stage of stress.

Endogenous hormones are key signaling molecules that regulate numerous core processes, including metabolism, growth, development, and reproduction. When plants encounter drought stress, hormone homeostasis is disrupted, triggering complex signaling cascades that initiate adaptive responses (Aliniaeifard et al., 2023; Thilakarathne et al., 2025). In this study, leaf IAA content decreased significantly under drought stress, accompanied by the accumulation of MeIAA and IAA-Glu, suggesting that the formation of conjugated forms may contribute to reduced IAA levels. DPC treatment was associated with higher leaf IAA contents and lower accumulation of MeIAA and IAA-Glu than drought treatment alone. Conversely, DPC treatment was associated with changes in TRP, TRA, MeIAA, IAA, and related auxin metabolites in roots under drought stress, resulting in increased precursor and IAA contents and thereby providing a basis for increases in total root length and root surface area. With respect to cytokinins, DPC significantly increased iPR levels in leaves while inhibiting the accumulation of its precursor, iPRMP. In roots, however, iPR was inhibited, whereas iPRMP accumulated markedly. This difference may occur because leaves maintain relatively high levels of active iPR to preserve basic cellular activity and delay leaf senescence, whereas limiting the conversion of iPRMP to iPR in roots may weaken local radial growth to some extent, thereby facilitating more efficient allocation of assimilates to the longitudinal elongation of primary and lateral roots (Li et al., 2006; Dong et al., 2008). Under drought stress, plants often accumulate hormones such as ABA and JA to initiate intracellular physiological defenses (Margay et al., 2024). However, the distribution of these hormones between roots and leaves directly determines the trade-off between survival and growth. In this study, ABA contents in the roots and leaves of both soybean cultivars increased significantly under drought stress, whereas DPC treatment significantly downregulated this effect. This may help alleviate damage to the photosynthetic apparatus caused by high ABA concentrations in leaves and reduce the inhibition of root elongation. Meanwhile, the increased contents of JA, JA-Ile, and OPDA in leaves after DPC treatment may be associated with altered jasmonate-related responses under drought stress. In roots, however, DPC treatment caused JA and its active form to show decreasing trends. Previous studies have shown that high ABA concentrations reduce auxin concentrations in root tips by inhibiting auxin biosynthesis and transport, ultimately inhibiting primary-root elongation, whereas JA inhibits primary-root elongation by suppressing root apical meristem activity (Chen et al., 2011; Luo et al., 2023). Therefore, maintaining relatively low ABA and JA levels in soybean roots under drought stress through DPC treatment may partly reduce the inhibition of root elongation, thereby creating a more favorable endogenous hormonal environment for root morphological development. The hormonal results further showed that the two cultivars differed in their hormone-related metabolite response patterns to DPC treatment under drought stress. Several hormone-related metabolites showed greater changes in HN44, whereas the responses of the corresponding metabolites were relatively weaker in HN65. Together with the contrasting response patterns observed between roots and leaves, these results suggest that DPC treatment was associated with cultivar- and tissue-dependent changes in hormone-related metabolites under drought stress.

Taken together, the results suggest that DPC-associated responses under drought stress were not limited to a single trait, but involved concurrent changes in plant morphology and stress-related physiology. Relatively restrained shoot growth, together with improved root development, may contribute to a balance between water consumption and water acquisition. At the same time, the maintenance of photosynthetic performance, lower oxidative damage, and enhanced antioxidant and osmotic-regulation responses may help sustain cellular functioning under drought stress. Increased phenylpropanoid-metabolism-related enzyme activities and lignin accumulation may further contribute to tissue structural stability. The distinct hormone-related metabolite responses observed between roots and leaves may be associated with these aboveground and belowground differences. Thus, the DPC-associated responses observed in this study provide an integrated physiological perspective on soybean adaptation to drought stress. Nevertheless, several limitations should be considered when interpreting these results. The responses to DPC treatment were evaluated in only two soybean cultivars and under a single experimental setting; therefore, the general applicability of the observed responses should be assessed across a broader range of genotypes and environmental conditions. In addition, the present study focused on morphological, physiological, biochemical, and hormone-metabolite responses. Further studies combining molecular analyses with measurements of hormone metabolism and transport would help clarify the regulatory basis of DPC-associated responses under drought stress.

## 5 Conclusions

Using two soybean cultivars with contrasting drought tolerance, this study systematically evaluated the regulatory effects of DPC on soybean yield formation and drought-stress alleviation. Field experiments showed that 200 mg L^-^¹ DPC was an appropriate application concentration and significantly improved soybean yield by optimizing plant architecture and pod-setting structure. Under drought stress, DPC induced coordinated responses characterized by the inhibition of excessive shoot expansion and the promotion of belowground root development, which may help reduce water consumption and enhance root water-uptake capacity. In addition, DPC maintained photosynthetic electron-transport efficiency in leaves, enhanced the activities of antioxidant enzymes, including SOD, POD, and APX, promoted the accumulation of osmotic-regulatory substances, and alleviated ROS accumulation and membrane lipid peroxidation damage. DPC also enhanced plant structural stability and stress adaptability by activating the phenylpropanoid metabolic pathway, promoting lignin synthesis, and reshaping endogenous hormone balance in roots and leaves.

## Data Availability

The original contributions presented in the study are included in the article/**Supplementary Material**. Further inquiries can be directed to the corresponding author.
