# Supplementary material for: Regulatory effects of mepiquat chloride on soybean yield formation and physiological responses to drought stress
Source: Front Plant Sci. 2026 Sep 7;17:1923691. doi: 10.3389/fpls.2026.1923691 (PMC13590336; doi:10.3389/fpls.2026.1923691)
Supplement: Supplementary file 1 [file Supplementaryfile1.docx]

# Supplementary Table 1. Basic soil fertility.

| Total Nitrogen  （g/kg） | Available Nitrogen (mg/kg) | Total Phosphorus (g/kg) | Available Phosphorus (mg/kg) | Total Potassium (g/kg) | Available Potassium (mg/kg) | pH | Organic Matter (g/kg) |
| --- | --- | --- | --- | --- | --- | --- | --- |
| 1.12 | 84.38 | 0.67 | 31.43 | 22.31 | 154.72 | 6.83 | 32.57 |

# Supplementary Table 2. Composition of nutrient solution

| Inorganic salt | Concentration（mg/L） | Inorganic salt | Concentration（mg/L） |
| --- | --- | --- | --- |
| MgSO₄ | 240.00 | CuSO₄·5H₂O | 0.08 |
| KH₂PO₄ | 136.00 | ZnSO₄·7H₂O | 0.22 |
| NH₄NO₃ | 142.86 | MnCl₂·4H₂O | 4.90 |
| CaCl₂ | 220.00 | H₃BO₃ | 2.86 |
| Na₂MoO₄·H₂O | 0.03 | Fe-EDTA | * |

Note: Dissolve 5.57 g FeSO₄·7H₂O and 7.45 g Na₂EDTA separately to 1 L, and add 1 mL of each stock solution per liter of nutrient solution.

# Supplementary Table 3. Multi-factor analysis of variance of blades

| Index | Cultivar(C) | | Treatment（T） | | Day（D） | | C × T | | C × D | | T× D | | C × T × D | |
| --- | --- | --- | --- | --- | --- | --- | --- | --- | --- | --- | --- | --- | --- | --- |
|  | F | P | F | P | F | P | F | P | F | P | F | P | F | P |
| POD | 58.66 | *** | 519.79 | *** | 231.91 | *** | 12.17 | *** | 11.22 | *** | 53.99 | *** | 1.93 | 0.13 |
| SOD | 675.72 | *** | 395.89 | *** | 230.80 | *** | 54.37 | *** | 271.04 | *** | 41.29 | *** | 4.42 | ** |
| APX | 22.17 | *** | 656.73 | *** | 561.79 | *** | 0.13 | 0.88 | 66.78 | *** | 152.08 | *** | 11.88 | *** |
| H_2_O_2_ | 171.77 | *** | 946.60 | *** | 509.88 | *** | 113.03 | *** | 26.81 | *** | 110.44 | *** | 13.21 | *** |
| MDA | 111.68 | *** | 253.94 | *** | 172.77 | *** | 20.53 | *** | 7.90 | ** | 27.94 | *** | 2.54 | 0.06 |
| pro | 18.30 | *** | 396.67 | *** | 355.62 | *** | 6.82 | ** | 5.41 | ** | 74.44 | *** | 2.25 | 0.08 |
| Ss | 235.99 | *** | 109.48 | *** | 371.02 | *** | 2.30 | 0.12 | 2.29 | 0.12 | 5.92 | ** | 1.63 | 0.19 |
| Sp | 2.67 | 0.11 | 164.80 | *** | 363.23 | *** | 8.48 | ** | 25.29 | *** | 11.32 | *** | 2.20 | 0.09 |
| PAL | 13.00 | ** | 157.81 | *** | 79.95 | *** | 2.46 | 0.10 | 13.74 | *** | 22.32 | *** | 0.97 | 0.44 |
| C4H | 127.54 | *** | 194.88 | *** | 105.80 | *** | 2.53 | 0.09 | 17.49 | *** | 10.46 | *** | 3.69 | * |
| CAD | 10.49 | ** | 261.41 | *** | 169.37 | *** | 2.00 | 0.15 | 25.18 | *** | 23.09 | *** | 2.84 | * |
| C4L | 340.29 | *** | 118.49 | *** | 88.67 | *** | 8.35 | ** | 23.68 | *** | 6.84 | *** | 4.03 | ** |
| Lignin content | 294.40 | *** | 106.43 | *** | 145.14 | *** | 8.47 | ** | 0.83 | 0.44 | 7.86 | *** | 0.51 | 0.73 |
| Dry Weight | 18.18 | *** | 386.34 | *** | 1,907.15 | *** | 1.44 | 0.25 | 6.73 | ** | 156.25 | *** | 2.25 | 0.08 |
| Fresh Weight | 1.74 | 0.20 | 487.39 | *** | 952.32 | *** | 6.05 | ** | 0.17 | 0.85 | 148.04 | *** | 3.23 | * |
| Plant height | 2.66 | 0.11 | 87.17 | *** | 1,148.06 | *** | 0.71 | 0.50 | 0.21 | 0.81 | 24.82 | *** | 1.36 | 0.27 |
| Plant Leaf-area | 8.04 | ** | 251.45 | *** | 708.09 | *** | 2.19 | 0.13 | 0.72 | 0.49 | 104.35 | *** | 3.13 | * |
| Stem diameter | 4.49 | * | 177.41 | *** | 190.98 | *** | 16.51 | *** | 6.49 | ** | 7.58 | *** | 6.32 | ** |
| Fv'/Fm' | 3.41 | 0.07 | 21.24 | *** | 12.32 | *** | 0.75 | 0.48 | 2.10 | 0.14 | 5.69 | ** | 1.08 | 0.38 |
| Phi2 | 41.45 | *** | 252.46 | *** | 406.55 | *** | 11.84 | *** | 21.51 | *** | 8.14 | *** | 12.13 | *** |
| SPAD | 40.43 | *** | 95.27 | *** | 76.13 | *** | 0.09 | 0.92 | 7.31 | ** | 6.95 | *** | 0.53 | 0.72 |
| NPQt | 0.23 | 0.64 | 372.33 | *** | 1,041.04 | *** | 7.50 | *** | 8.42 | ** | 93.83 | *** | 5.09 | ** |

# Note: C, T, and D denote the main effects of Cultivar, Treatment and Day, respectively. C × T, C × D, T × D and C × T × D represent their interaction effects. F represents the F-statistic value from the ANOVA. Asterisks indicate statistical significance: * p < 0.05, ** p < 0.01, and *** p < 0.001. Values without asterisks indicate no significant difference (p ≥ 0.05), as in the following tables.

# Supplementary Table 4. Multivariate Analysis of Variance for Root Systems

| Index | Cultivar(C) | | Treatment（T） | | Day（D） | | C × T | | C × D | | T× D | | C × T × D | |
| --- | --- | --- | --- | --- | --- | --- | --- | --- | --- | --- | --- | --- | --- | --- |
|  | F | P | F | P | F | P | F | P | F | P | F | P | F | P |
| POD | 0.57 | 0.46 | 153.15 | *** | 5.95 | ** | 8.16 | ** | 1.45 | 0.25 | 2.91 | * | 0.70 | 0.60 |
| SOD | 4.38 | * | 61.78 | *** | 70.24 | *** | 5.74 | ** | 7.59 | ** | 14.47 | *** | 2.93 | * |
| APX | 13.82 | ** | 86.31 | *** | 36.46 | *** | 3.60 | * | 21.96 | *** | 2.38 | 0.07 | 3.87 | ** |
| H_2_O_2_ | 1,742.22 | *** | 8,858.63 | *** | 3,384.02 | *** | 977.20 | *** | 476.42 | *** | 1,337.90 | *** | 365.10 | *** |
| MDA | 37.29 | *** | 434.11 | *** | 228.24 | *** | 19.36 | *** | 18.65 | *** | 64.65 | *** | 6.05 | ** |
| Pro | 34.88 | *** | 155.37 | *** | 170.04 | *** | 2.62 | 0.09 | 7.47 | ** | 24.67 | *** | 2.27 | 0.08 |
| Ss | 18.83 | *** | 78.78 | *** | 99.06 | *** | 4.06 | 0.03 | 19.62 | *** | 5.74 | ** | 0.11 | 0.98 |
| Sp | 0.29 | 0.59 | 165.97 | *** | 74.11 | *** | 6.01 | ** | 2.05 | 0.14 | 2.06 | 0.11 | 2.24 | 0.08 |
| PAL | 9.49 | ** | 148.72 | *** | 41.34 | *** | 5.30 | * | 7.69 | ** | 23.27 | *** | 3.30 | * |
| C4H | 1,506.30 | *** | 217.22 | *** | 196.87 | *** | 43.07 | *** | 40.17 | *** | 36.66 | *** | 22.58 | *** |
| CAD | 10.10 | ** | 415.01 | *** | 402.02 | *** | 27.82 | *** | 5.51 | ** | 36.71 | *** | 15.98 | *** |
| 4CL | 11.58 | ** | 85.68 | *** | 158.04 | *** | 4.75 | * | 39.07 | *** | 20.61 | *** | 2.60 | * |
| Lignin content | 71.58 | *** | 119.92 | *** | 301.97 | *** | 0.20 | 0.82 | 21.58 | *** | 10.80 | *** | 1.12 | 0.36 |
| Dry Weight | 17.99 | *** | 384.47 | *** | 1,899.98 | *** | 1.44 | 0.25 | 6.67 | ** | 155.55 | *** | 2.27 | 0.08 |
| Fresh Weight | 1.74 | 0.20 | 487.36 | *** | 952.25 | *** | 6.03 | ** | 0.17 | 0.85 | 148.02 | *** | 3.24 | * |
| Total root length | 19.77 | *** | 80.69 | *** | 316.33 | *** | 0.57 | 0.57 | 4.11 | * | 51.36 | *** | 0.20 | 0.94 |
| Root volume | 148.75 | *** | 156.90 | *** | 560.41 | *** | 66.33 | *** | 30.75 | *** | 142.85 | *** | 24.85 | *** |
| Root surface area | 31.52 | *** | 147.13 | *** | 396.13 | *** | 1.02 | 0.37 | 24.19 | *** | 32.53 | *** | 3.11 | * |

# Supplementary Table 5. Multivariate analysis of variance on hormone content in soybean leaves and roots

|  | Indices | Cultivar(C) | | Treatment（T） | | C × T | |
| --- | --- | --- | --- | --- | --- | --- | --- |
|  |  | F | P | F | P | F | P |
| Leaf | ABA | 87.059 | *** | 340.070 | *** | 23.145 | *** |
|  | TRP | 1.288 | 0.279 | 50.466 | *** | 2.165 | 0.157 |
|  | TRA | 15.329 | ** | 10.443 | ** | 1.652 | 0.232 |
|  | MeIAA | 152.066 | *** | 17.077 | *** | 3.669 | * |
|  | IAA | 29.411 | *** | 9.653 | ** | 1.834 | 0.202 |
|  | IAA-Asp | 50.794 | *** | 9.016 | ** | 0.643 | 0.543 |
|  | IAA-Glu | 6.496 | * | 9.228 | ** | 0.593 | 0.568 |
|  | iPRMP | 10.673 | ** | 10.923 | ** | 0.889 | 0.436 |
|  | iPR | 27.330 | *** | 5.891 | * | 1.885 | 0.194 |
|  | OPDA | 68.767 | *** | 44.982 | *** | 15.551 | *** |
|  | JA | 201.761 | *** | 24.337 | *** | 2.402 | 0.133 |
|  | JA-ILE | 313.217 | *** | 2.260 | 0.147 | 0.485 | 0.627 |
|  | ABA | 0.166 | 0.691 | 9.612 | ** | 1.768 | 0.212 |
|  | TRP | 2.836 | 0.118 | 23.286 | *** | 1.599 | 0.242 |
| Root | TRA | 7.883 | 0.016 | 31.646 | *** | 1.568 | 0.248 |
|  | MeIAA | 14.544 | ** | 83.293 | *** | 4.558 | * |
|  | IAA | 41.670 | *** | 90.698 | *** | 10.358 | *** |
|  | IAA-Asp | 17.792 | ** | 277.484 | *** | 42.931 | *** |
|  | IAA-Glu | 806.270 | *** | 575.077 | *** | 279.601 | *** |
|  | iPRMP | 5.777 | * | 196.719 | *** | 5.472 | * |
|  | iPR | 13.345 | ** | 32.407 | *** | 9.832 | ** |
|  | OPDA | 63.724 | *** | 27.224 | *** | 2.066 | 0.169 |
|  | JA | 6.300 | * | 208.851 | *** | 7.303 | ** |
|  | JA-ILE | 3.354 | 0.092 | 71.005 | *** | 4.353 | * |
