# Supplementary material for: Regulatory effects of mepiquat chloride on soybean yield formation and physiological responses to drought stress
Source: Front Plant Sci. 2026 Sep 7;17:1923691. doi: 10.3389/fpls.2026.1923691 (PMC13590336; doi:10.3389/fpls.2026.1923691)
Supplement: Supplementary file 2 [file Supplementaryfile2.docx]

Fig. S1. Daily meteorological conditions during the soybean field experiment from May 1 to October 15, 2024.

Note: (A) Daily minimum air temperature (Min Temp), mean air temperature (AVG Temp), and maximum air temperature (Max Temp). (B) Daily precipitation. Bars indicate measurable precipitation amount (mm), whereas short horizontal lines indicate trace precipitation events, for which precipitation was observed but no measurable amount was recorded.
